# Dual-Atom Nanozyme Eye Drops Attenuate Inflammation and Break the Vicious Cycle in Dry Eye Disease

**DOI:** 10.1007/s40820-024-01322-7

**Published:** 2024-02-19

**Authors:** Dandan Chu, Mengyang Zhao, Shisong Rong, Wonho Jhe, Xiaolu Cai, Yi Xiao, Wei Zhang, Xingchen Geng, Zhanrong Li, Xingcai Zhang, Jingguo Li

**Affiliations:** 1 Henan Eye Hospital, Henan Provincial People’s Hospital, People’s Hospital of Zhengzhou University, Zhengzhou, 450003 People’s Republic of China; 2 Department of Ophthalmology, Mass Eye and Ear, Mass General Brigham, Harvard Medical School, Boston, MA 02114 USA; 3 School of Engineering and Applied Sciences, Harvard University, Cambridge, MA 02138 USA; 4 College of Chemistry, Zhengzhou University, Zhengzhou, 450001 People’s Republic of China

**Keywords:** Dry eye disease, DAN, Dual-atom nanozyme, Vicious cycle, NLRP3 inflammasome, Nanomedicine

## Abstract

Dry eye disease (DED) is a major ocular pathology worldwide, causing serious ocular discomfort and even visual impairment. The incidence of DED is gradually increasing with the high-frequency use of electronic products. Although inflammation is core cause of the DED vicious cycle, reactive oxygen species (ROS) play a pivotal role in the vicious cycle by regulating inflammation from upstream. Therefore, current therapies merely targeting inflammation show the failure of DED treatment. Here, a novel dual-atom nanozymes (DAN)-based eye drops are developed. The antioxidative DAN is successfully prepared by embedding Fe and Mn bimetallic single-atoms in N-doped carbon material and modifying it with a hydrophilic polymer. The in vitro and in vivo results demonstrate the DAN is endowed with superior biological activity in scavenging excessive ROS, inhibiting NLRP3 inflammasome activation, decreasing proinflammatory cytokines expression, and suppressing cell apoptosis. Consequently, the DAN effectively alleviate ocular inflammation, promote corneal epithelial repair, recover goblet cell density and tear secretion, thus breaking the DED vicious cycle. Our findings open an avenue to make the DAN as an intervention form to DED and ROS-mediated inflammatory diseases.

**Supplementary Information:**

The online version contains supplementary material available at 10.1007/s40820-024-01322-7.

## Highlights

- A dual-atom nanozyme (DAN) was successfully prepared based on Fe and Mn bimetallic single-atom embedded in N-doped carbon material and modified with hydrophilic polymer.
- The DAN possess excellent enzyme catalytic activity and attenuate dramatically inflammation by inhibiting the reactive oxygen species (ROS)/NLRP3 signal axis.
- The DAN break the vicious cycle in dry eye disease and is a potential strategy for treating dry eye disease.

**Supplementary Information:**

The online version contains supplementary material available at 10.1007/s40820-024-01322-7.

## Graphical abstract

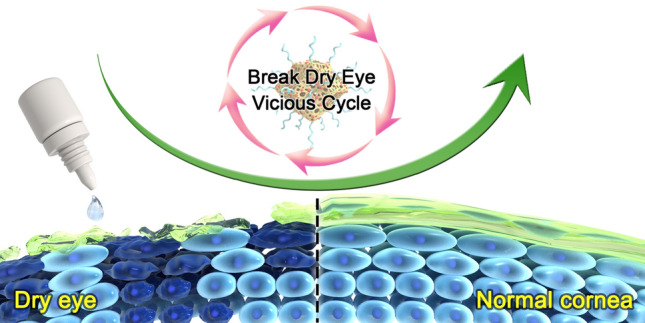

## Introduction

The increasingly prevalence of dry eye disease (DED) constitutes a significant global health concern, adversely impacting ocular comfort and quality of life. This trend is exacerbated by the widespread use of electronic products and sedentary lifestyles. DED is a multifactorial disease which could result in ocular dryness, discomfort, irritation, burning, itching, pain, and visual impairment [1, 2]. Dry eye vicious cycle refers to a self-perpetuating sequence of events including hyperosmolarity, oxidative stress damage, ocular surface inflammation, dysfunction and apoptosis, and tear film instability. In other words, initiated by tear film instability and subsequent hyperosmolarity, this cycle activates cellular stress pathways, leading to the secretion of several pro-inflammatory mediators such as IL-1β and IL-18. These mediators recruit additional inflammatory cells to the ocular surface, further escalating the inflammatory response [3]. The combined effects of these mediators and tear hyperosmolarity result in reduced glycocalyx mucin expression, apoptosis of surface epithelial cells, and loss of goblet cells, a hallmark of all forms of DED [4]. Furthermore, hyperosmolarity induces non-apoptotic corneal epithelial cell death. Changes in glycocalyx mucins likely contribute to ocular surface staining in DED and impair ocular surface humidity, leading to early tear film breakup. This exacerbates ocular surface hyperosmolarity, thus perpetuating the disease through a “vicious cycle”. This cycle aggravates DED symptoms, complicates its consequences, and challenges treatment efficacy.

Effective management of DED requires a multimodal approach aimed at disrupting multiple aspects of the self-perpetuating cycle. The complexity of the vicious cycle often impedes the effectiveness of current treatment options. While inflammation, stemming from both early innate and subsequent adaptive immune responses, is a critical element in the vicious cycle of DED [1–4], existing anti-inflammatory treatments exhibit variable efficacy and carry risks such as glaucoma and cataract from corticosteroids, and ocular burning from cyclosporine A (CsA) [5]. Furthermore, these treatments generally necessitate repeated administration at high concentrations, owing to rapid ocular surface drug clearance. This can lead to side effects like transient reduced vision and ocular discomfort, adversely affecting patient compliance. Consequently, more effective and safer therapies for DED is an active area of research.

In exploring new treatments for DED, targeting oxidative stress has emerged as a viable approach. Excessive oxidative stress, known to activate inflammatory responses, contributes significantly to DED pathogenesis [6, 7]. Oxidative stress in DED results from an imbalance between reactive oxygen species (ROS) production and their detoxification, often due to factors like tear hyperosmolarity [7–9] or aging [10]. Elevated ROS levels can activate inflammatory pathway. Firstly, ROS activate the nuclear transcription factor-κB (NF-κB), leading to phosphorylation and upregulate the expression of pro interleukin-1β (IL-1β) and interleukin-18 (IL-18). Secondly, ROS induce inflammation by inflammasomes pathway. Inflammasomes are multiprotein complexes localized in the cytoplasm of the cell that play a crucial role in innate immunity, which are assembled, activated, and mediated the occurrence of inflammation [11, 12]. The most widely studied and fully characterized member of the identified inflammasomes is NLRP3 inflammasome, which composed of NLRP3 (nucleotide-binding domain leucine-rich repeat (NLR) and pyrin domain containing receptor 3), ASC (apoptosis-associated speck-like protein containing a C-terminal caspase recruitment domain), and pro-caspase-1 [13, 14]. ROS activates the NLRP3 inflammasome and causes cascade reactions to promote the secretion of IL-1β and IL-18, resulting in ocular surface inflammation and forming a “vicious cycle” in dry eye [15, 16]. Conversely, inflammation exacerbates oxidative stress through the release of inflammatory mediators, perpetuating a vicious cycle. Moreover, ROS causes direct damage to cellular components such as lipids, proteins, and DNA, triggering inflammatory responses needed for cellular repair [17]. Therefore, ROS, as an upstream signal of inflammation, is a key target for regulating inflammation. Although inhibiting NLRP3 activation via ROS reduction is a proposed dry eye treatment, the efficacy of this approach remains to be optimized [18–22]. Currently dual-atom nanozyme (DAN) have the superior potent of scavenging excess ROS. Therefore, topical DAN that targeting ROS might benefit in efficiently controlling the inflammation in DED.

Nanozymes are nanomaterials with enzyme-like activity, which can efficiently catalyze the reaction of substrates under physiological conditions and follows similar kinetics and mechanism as natural enzymes [23–28]. Scientists have proposed that the integrated synthesis of Single-atom nanozymes (SAzymes) based on advanced single-atom catalysis technology and the active site of innate enzymes is a promising strategy for developing high-performance nanozymes [29]. SAzymes has unique characteristics such as clear atomic structure, electronic coordination environment, maximum atomic utilization rate, etc., so it has higher catalytic activity, stability, and extremely high selectivity [29–31]. SAzymes modelled the catalytic processes of natural enzymes such as peroxidase (POD), catalase (CAT), oxidase (OXD), superoxide dismutase (SOD) and glutathione peroxidase (GPx) to regulate the level of ROS in cells and the microenvironment of the pathological region, thus regulating the production of ROS [32]. To further improve the catalytic activity of SAzymes, new coordination strategies [33–35] that can reduce the amount of catalyst, change the electronic structure of the active site, regulate the binding energy, and achieve the optimal metal state between the catalytic active site and the reactants, intermediates, and products are promising and may have better effects on cellular oxidative stress and antiinflammation.

**Scheme 1:**
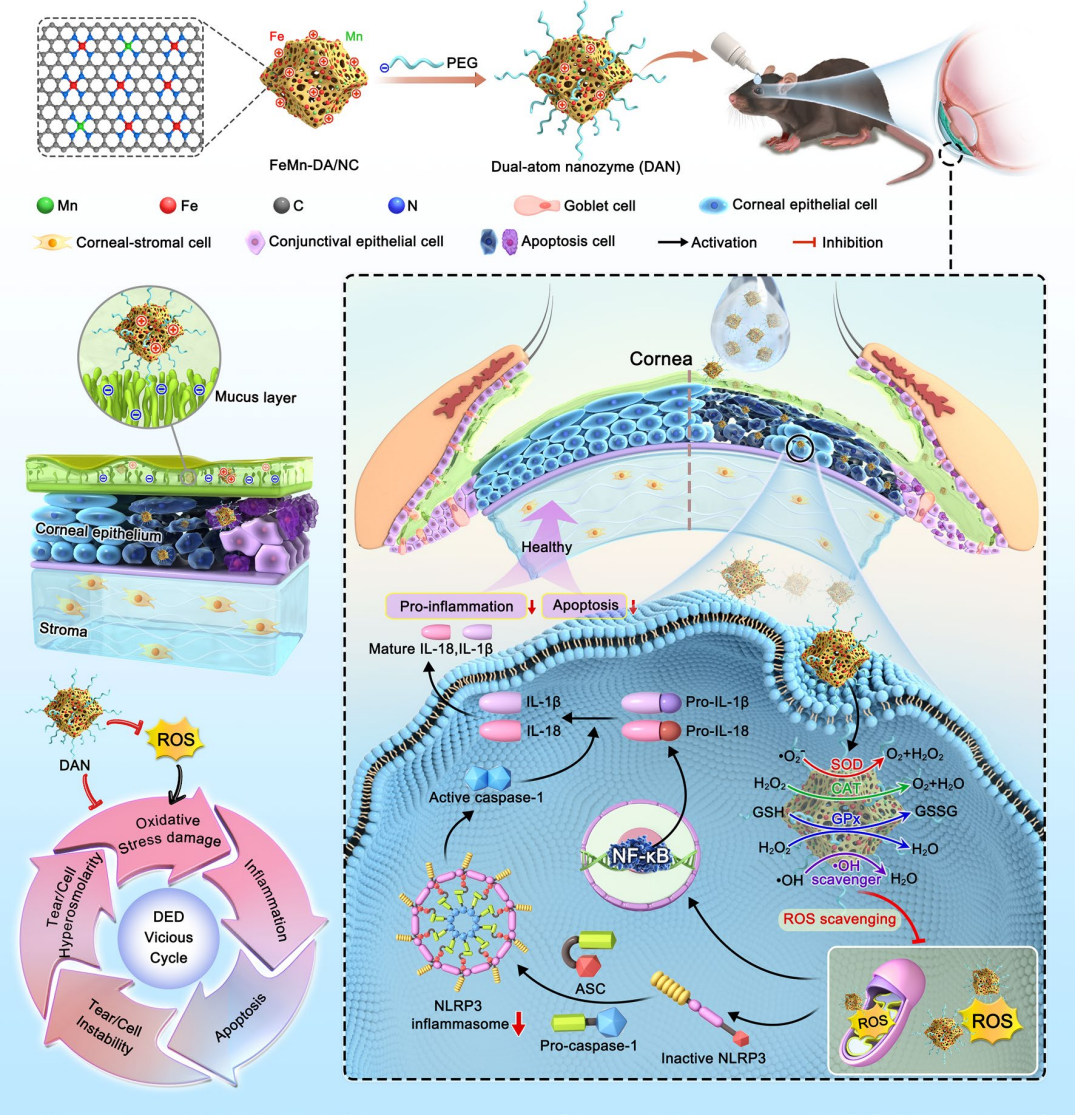
Schematic diagram of the synthesis of dual-atom nanozyme and break the DED vicious cycle by inhibiting NLPR3 inflammasome activation

In this study, we introduced a DAN eye drop formulation, FeMn-DAN, designed to inhibit NLPR3 inflammasome activation and mitigate ocular inflammation, disrupting the vicious cycle in DED (Scheme 1). Fe and Mn atoms are dispersed in single-atom state on the surface of N-doped carbon materials with pointed dodecahedron structure. The bimetallic synergy between Fe and Mn effectively neutralizes excess ROS in the ocular surface microenvironment, thereby reducing oxidative stress and direct oxidative damage. Thus, the current research was designed to investigate the role of DAN and the potential mechanisms in DED.

## Experimental and Methods

### Preparation of DAN

FeMnZIF-8 nanocrystals were synthesized according to previous studies, with some modifications. Zn(NO_3_)_2_·6H_2_O (1.601 g), MnCl_2_ (0.0389 g), and Fe(acac)_3_ (0.7 g) were dissolved in methanol (80 mL). A mixture of 2-methylimidazole (3.7 g) and methanol (80 mL) was added to the above solution, and the mixture was vigorously stirred for 24 h at room temperature. Subsequently, the obtained raw FeMnZIF-8 was collected by centrifugation and washed twice with methanol. The resulting precipitate was further treated at 120 °C in a vacuum for 24 h before further use.

The synthesized FeMnZIF-8 powder was dispersed in methanol (120 mL) by ultrasonication. The dispersion solution was then mixed with H_2_O (120 mL), followed by the introduction of CTAB (25 mg mL^−1^) and NaOH (6 mg mL^−1^). TEOS (1.2 mL) was then injected and the mixture was stirred at room temperature. After 2 h, the particles were collected by centrifugation and washed twice with ethanol. The final product was named FeMnZIF-8@SiOx and dried under vacuum for further use.

FeMnZIF-8@SiOx was placed in a temperature-programmed furnace under Ar flow. The temperature was increased at 5 °C min^−1^ to 300 °C and remained at this temperature for 2 h. Then, the calcining temperature was further increased to 900 °C at the same ramp rate and remained at this temperature for 5 h to obtain FeMn-DA/NC@SiOx. Finally, the product was naturally cooled to room temperature.

The resulting products were washed three times with 12% HF, 1 M HCl, H_2_O, and then methanol, and dried at 60 °C under vacuum to yield FeMn-DA/NC.

FeMn-DA/NC (5 mg) and PEG_1000_ (20 mg) were stirred in the water for 12 h at room temperature. And then the unreacted PEG_1000_ was removed by centrifugation and washing. After redispersion, hydrophilic DAN was acquired and the DAN eye drops were obtained.

### In vitro Antioxidative Properties

Tear hyperosmolality is one of the main characteristics of DED, leading to ROS overproduction, which triggers the vicious cycle of DED development. In HCE-2 cells, hypertonic conditions (500 mOsM) was applied to generate excess ROS [15]. HCE-2 cells intracellular ROS generation was detected using 2′,7′-dichlorofluorescein diacetate (DCFH-DA). Briefly, human corneal epithelial cells (HCE-2) were seeded in six-well plates at the same density and incubated overnight at 37 °C under 5% CO_2_ conditions. Then the cells were pretreated with serum-free DMEM/F12 medium containing different concentrations of DAN (1, 2, 4, and 8 µg mL^−1^) for 6 h. Subsequently, the cells were cultured in serum-free DMEM-F12 containing 90 mM NaCl, which was used to create a hypertonic environment (500 mOsM) for an additional 24 h. The ROS inhibitor NAC (10 mM) was added 1 h before NaCl supplementation as a positive control. Cells were washed twice with PBS and stained for 30 min at 37 °C with 10 µM DCFH-DA in the dark. Cells were cultured in physiological isotonic conditions were used as control. Each group was observed and photographed under a fluorescence microscopy (OlympusCKX41SF, Japan).

To further determine the ability of DAN to scavenge mitochondria-specific ROS, the mitochondrial ROS production of HCE-2 cells received various treatments were studied. HCE-2 cells were seeded onto confocal culture dishes (35 mm, Biosharp, China) and incubated for 24 h in DMEM/F12 complete medium. Then, the cells were pre-treated with serum-free DMEM/F12 medium containing DAN (2 µg mL^−1^) for 6 h and incubated with hypertonic medium (500 mOsM) for 24 h. The cells were washed twice with PBS and stained with DAPI solution (10 µg mL^−1^), a mitochondrial tracker (200 nM, MitoTracker™ Green FM) and a mitochondrial superoxide indicator (2 μM, MitoSOX™ Red) for 20 min. HCE-2 cells were observed and photographed under a confocal laser scanning microscopy (CLSM, NikonC1Si, Japan).

To evaluate the antioxidant enzyme activity of DAN, immunofluorescence analysis of SOD1, GPX1, CAT and HO-1 were performed. HCE-2 cells were seeded onto coverslips that had been placed into the wells of a six-well plate at a density of 5 × 10^3^ cells per well and administrated with DAN (2 μg mL^−1^) as above mentioned. After the cells were fixed with 4% paraformaldehyde (Biosharp, China) for 10 min, they were washed and permeabilized with 0.5% Triton for 20 min and then blocked with goat serum (Elabscience, China) for 30 min at room temperature. Then, the HCE-2 cells were incubated with primary antibodies against SOD1, GPX1, CAT and HO-1 overnight at 4 °C. The next day, cells were incubated with FITC-labeled goat anti-rabbit IgG H&L conjugated secondary antibody in the dark for 1 h at room temperature, and the nuclei were counterstained with DAPI. Images were acquired by fluorescence microscopy (Nikon ECLIPSE 80i, Japan).

HCE-2 cells were seeded onto 10 cm culture dishes at a 5 × 10^6^ cells density and incubated for 24 h in DMEM/F12 complete medium. Then, the cells were pre-treated with serum-free DMEM/F12 medium containing DAN (2 µg mL^−1^) for 6 h and incubated with hypertonic medium (500 mOsM) for 24 h. The cells were harvested and counted. 1 ml of the extract was added per 1000 × 10^4^ cells and the cells were sonicated under ice bath conditions (200 W, 10 min). Then the above cell extracts were centrifuged (4 °C, 8000 g, 10 min) and the supernatant was taken and placed on ice for measurement. Subsequently, SOD, GPX and CAT activities in each group were detected using the appropriate assay kits according to the manufacturer’s instructions.

### In vitro Anti-Apoptotic Properties

8-OHdG and JC-1 immunofluorescence staining were performed to evaluate the anti-apoptotic effect of DAN. Briefly, HCE-2 cells were pre-treated with DAN for 6 h and then incubated with hypertonic medium (500 mOsM) for 24 h. Next, the cells were fixed, permeabilized and blocked with goat serum. Then cells were incubated with primary rabbit anti-8-OHdG (DNA/RNA Damage) antibody at 4 °C overnight. After washed three times with PBS, cells were incubated with FITC-labeled goat anti-rabbit IgG H&L conjugated secondary antibody for 1 h and stained with DAPI. The stained slides were monitored under a fluorescent microscope. In addition, DAN-pretreated and hypertonicity-treated HCE-2 cells were stained with JC-1 to detect changes in mitochondrial membrane potential according to the manufacturer’s instructions.

### In vitro Anti-Inflammatory Properties

To assess the anti-inflammatory effect of DAN on inhibiting NLRP3 inflammasome activation by mitigating oxidative stress, HCE-2 cells were pretreated with or without DAN in the hypertonic solution. HCE-2 cells were washed three times with PBS, fixed in 4% paraformaldehyde for 10 min at room temperature, washed with PBS three times, permeabilized with 0.5% Triton for 20 min, washed with PBS, and blocked with goat serum for 30 min at room temperature. Subsequently, primary antibodies against NLRP3, ASC, Caspase-1, IL-1β, IL-18, IL-6, NF-κB P65, P- NF-κB P65 were added and incubated overnight at 4 °C. Then, the cells were incubated with FITC-labeled goat anti-rabbit IgG H&L and Cy3-labeled goat anti-mouse IgG (H + L) secondary antibodies for 1 h at 37 °C. Nuclei were counterstained with DAPI. Images were captured by fluorescence microscopy.

### Western Blot Analysis

HCE-2 cells were pretreated with DAN for 6 h in advance and incubated with hypertonic medium (500 mOsM) to determine ROS/NLRP3/IL-1β signaling pathway protein expression. Proteins were extracted using RIPA buffer, and the protein concentrations were measured using a BCA protein assay kit. Equal amounts of protein samples were separated by 12.5% SDS-PAGE and electro transferred to PVDF membranes. The membranes were blocked with 5% nonfat dry milk at room temperature for 1 h and then probed with the following antibodies overnight at 4 °C: SOD1, GPX1, CAT, HO-1, NLRP3, ASC, Caspase-1, IL-1β, IL-18, IL-6, NF-κB P65, P- NF-κB P65 and β-actin. The membranes were then exposed to goat anti-rabbit-IgG-HRP and goat anti-mouse IgG-HRP for 1 h at room temperature, followed by imaging with a chemiluminescent substrate. The signals were analyzed using Image Lab and quantified using ImageJ.

### In vivo Therapeutic Efficacy Assessment on DED

#### Animal Models of DED

All experimental procedures for animal experiments were performed in accordance with the guidelines of the Chinese Animal Administration and the guidelines of the Association for Research in Vision and Ophthalmology Statement for the Use of Animals in Ophthalmic and Vision Research. Adult C57BL/6 female mice (6–8 weeks, 20–25 g) were used for animal studies and kept on a 12 h light/dark cycle at room temperature with enough food and water. 0.2% benzalkonium chloride (BAC, Hubei Gedian Humanwell Pharmaceutical Excipients Co., Ltd.) was used to create experimental DED model in mice. Briefly, each right eye of the mice was administered with 5 μL of 0.2% BAC eye drops (w/v) twice per day for 7 consecutive days. Then, the mice were randomly divided into three groups (n = 6 per group) and treated 5 μL topical eye drop solutions of 0.9% sterile saline (control), 100 μg mL^−1^ of DAN and 0.05% Cyclosporine A (CsA, Shenyang Xingqi Pharmaceutical Co., Ltd.) to the ocular surface twice per day (8 AM and PM) for 7 days. CsA was used as a positive control.

#### Tear Secretion Assessment

Tear secretion were measured using cotton threads (Liaoning Meizilin Pharmaceutical Co., Ltd.) according to the instruction. The threads were placed on the lower eyelid palpebral conjunctiva at 1/3 of the distance from the lateral canthus. The length of the wetted cotton thread was determined as the millimeters at 20 s without anesthesia.

#### Corneal Opacity and Fluorescein Staining

Corneal opacity was scored using a scale of 0–4 (0 = completely clear, 1 = slightly hazy, iris and pupils easily visible, 2 = slightly opaque, iris and pupils still detectable, 3 = opaque, pupils hardly detectable, and 4 = completely opaque with no view of the pupils). Corneal epithelial damage was evaluated using corneal fluorescein staining after sodium fluorescein was instilled into the conjunctival sac, and cornea was observed under the slit-lamp microscope with a cobalt blue filter. The cornea was divided into five areas (central, nasal, temporal, superior, and inferior), and each area was scored as follows: 0 = no staining, 1 = slight punctate staining, 2 = distinct punctate or slight coalescent staining, 3 = distinct coalescent or slight patchy staining, and 4 = distinct patchy staining [36, 37].

#### Histopathological Analysis

After the animals were sacrificed by overdose anesthesia, the eyes and adnexa were fixed in FAS eyeball fixative (Servicebio, China) and embedded in paraffin, cut into sagittal sections (5 μm thick), and then stored at room temperature. Eye sections were stained with hematoxylin and eosin (H&E) for routine histological analysis. Periodic acid Schiff (PAS) staining was used to observe and calculate conjunctival goblet cell number. TUNEL (a marker of apoptosis) immunofluorescence staining was performed using a TUNEL staining kit. The expression of ROS in corneal tissues was determined using Dihydroethidium. The expression of 8-OHdG, NLRP3, ASC, Caspase-1, IL-1β, IL-18 in corneal tissues were evaluated by immunofluorescence analysis. The stained sections were photographed with a digital light microscope (3D HISTECH P250 FLASH, Hungary).

### Statistical Analysis

GraphPad Prism Software Version 6.0 (GraphPad Software Inc., La Jolla, CA) was used to conduct all statistical analyses. All experiments were repeated at least three times, and the differences between groups were determined by one-way ANOVA. Differences were considered statistically significant at *p* < 0.05.

## Results and Discussion

### Characterization of FeMn-DA/NC

**Fig. 1:**
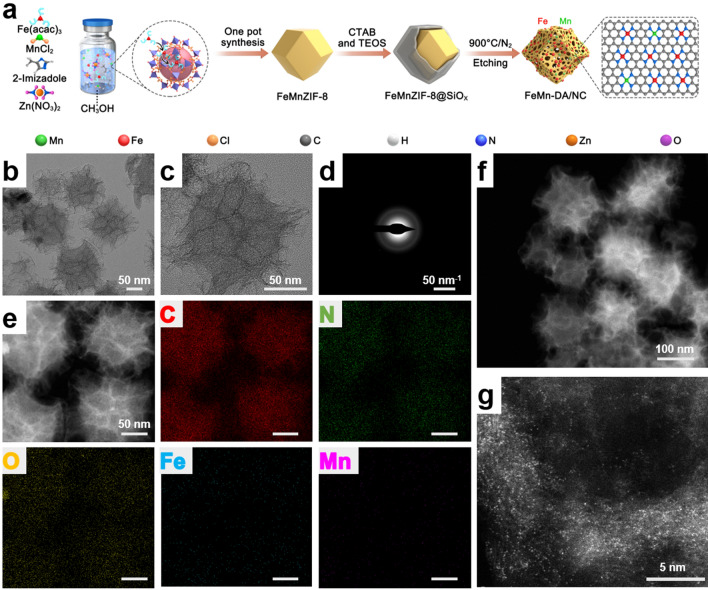
Synthetic procedure, morphology, and microstructure of FeMn-DA/NC. **a** Synthetic schematic diagram of FeMn-DA/NC. **b, c** Transmission electron microscope (TEM) image and **d** SAED image of FeMn-DA/NC. **e** Corresponding energy dispersive spectroscopy (EDS) mappings of FeMn-DA/NC, where C (red), N (green), O (yellow), Fe (cyan), and Mn (amaranth) were imaged under the STEM mode. **f, g** ac HAADF-STEM images of FeMn-DA/NC (isolated bright dots may be pairs of Fe and Mn single-atoms)

FeMn-DA/NC was prepared by a four-step method (Fig. 1a). Firstly, Fe(acac)_3_ provides iron atom and Mn come from MnCl_2_, are encapsulated with zeolite imidazole acid skeleton-8 (ZIF-8) with suitable nanocavity (11.6 and 3.4 Å) to form the trimetallic FeMnZIF-8. Compared with the X-ray diffraction (XRD) results of ZIF-8, it was found that FeMnZIF-8 was successfully prepared (Fig. S4). Secondly, the surface of FeMnZIF-8 is covered with a layer of SiOx to prevents irreversible fusion and aggregation during high-temperature pyrolysis. Then, the prepared FeMnZIF-8@SiOx is calcined under Ar flow to form Fe and Mn-single atoms on the N-doped carbon carrier (FeMn-DA/NC@SiOx). Finally, the SiOx shell and metal nanoparticles were removed with 12% hydrofluoric acid and 1 M hydrochloric acid, respectively, to form FeMn-DA/NC.

TEM and High-angle annular dark field scanning transmission electron microscopy with spherical aberration correction (ac HAADF-STEM) images show that FeMn-DA/NC is evenly distributed with an average plane size of 150 nm. FeMn-DA/NC retains the regular dodecahedron structure of ZIF-8, forming a porous carbon structure and a convex eaves shape, with rich edge shapes (Fig. 1b, c). The porous carbon structure and suspended structure are conducive to binding with catalytic substrates. The SEM images further confirm this result (Fig. S1). No single crystal or polycrystalline diffraction was observed in the selected region electron diffraction (SAED) image (Fig. 1d). The energy dispersive X-ray energy spectrum (EDS) further reveals the uniform distribution of C, N, O, Fe, and Mn (Figs. 1e and S2, S3). The uniform placement of Fe and Mn atoms in the structure indicates the generation of Fe and Mn double sites in the three-dimensional matrix. The ac-HAADF-STEM image confirms that the separated Fe and Mn atoms are anchored on n-doped carbon matrix carriers (Fig. 1f, g). The dispersion of small bright spots in FeMn-DA/NC is represented, which may be a single Fe atom, a single Mn atom or a double active site of FeMn. The results indicate that there is no dispersion of Fe and Mn nanoparticles on the N-doped carbon carrier. In addition, the XRD spectra of FeMn-DA/NC show peaks located at approximately 25° and 43°, respectively, belonging to the (002) and (101) faces of graphite (Fig. S4) [38]. X-ray photoelectron spectroscopy (XPS) analysis showed that FeMn-DA/NC was mainly composed of C, N, and O (Figs. S5–S7). The subpeaks at 285.6 and 288.9 eV in the C 1*s* region indicated that the SACs contained hydroxyl (C–OH), carbonyl (C=O), and C=N (Fig. S5). The O 1*s* spectrum is deconvoluted into three peaks at 532.0, 533.7, and 534.7 eV, attributed to the C=O, and –OH (Fig. S6). The presence of O can be ascribed to the adsorbed O-containing functional groups [39]. The high-resolution N 1*s* spectrum of FeMn-DA/NC in Fig. S7 can be deconvoluted into three peaks of graphite-N at 404.5 eV, pyrrolidine-N at 400.7 eV, and pyridine-N at 398.4 eV. Pyridine-N may include the contributions of Fe–N and Mn–N bonds. No significant Fe 2*p* and Mn 2*p* peaks are detected owing to the low content of Fe and Mn [40]. The content of Fe and Mn in FeMn-DA/NC measured by inductively coupled plasma mass spectrometry (ICP-MS) is 0.23 wt% and 0.02 wt%, respectively.

**Fig. 2:**
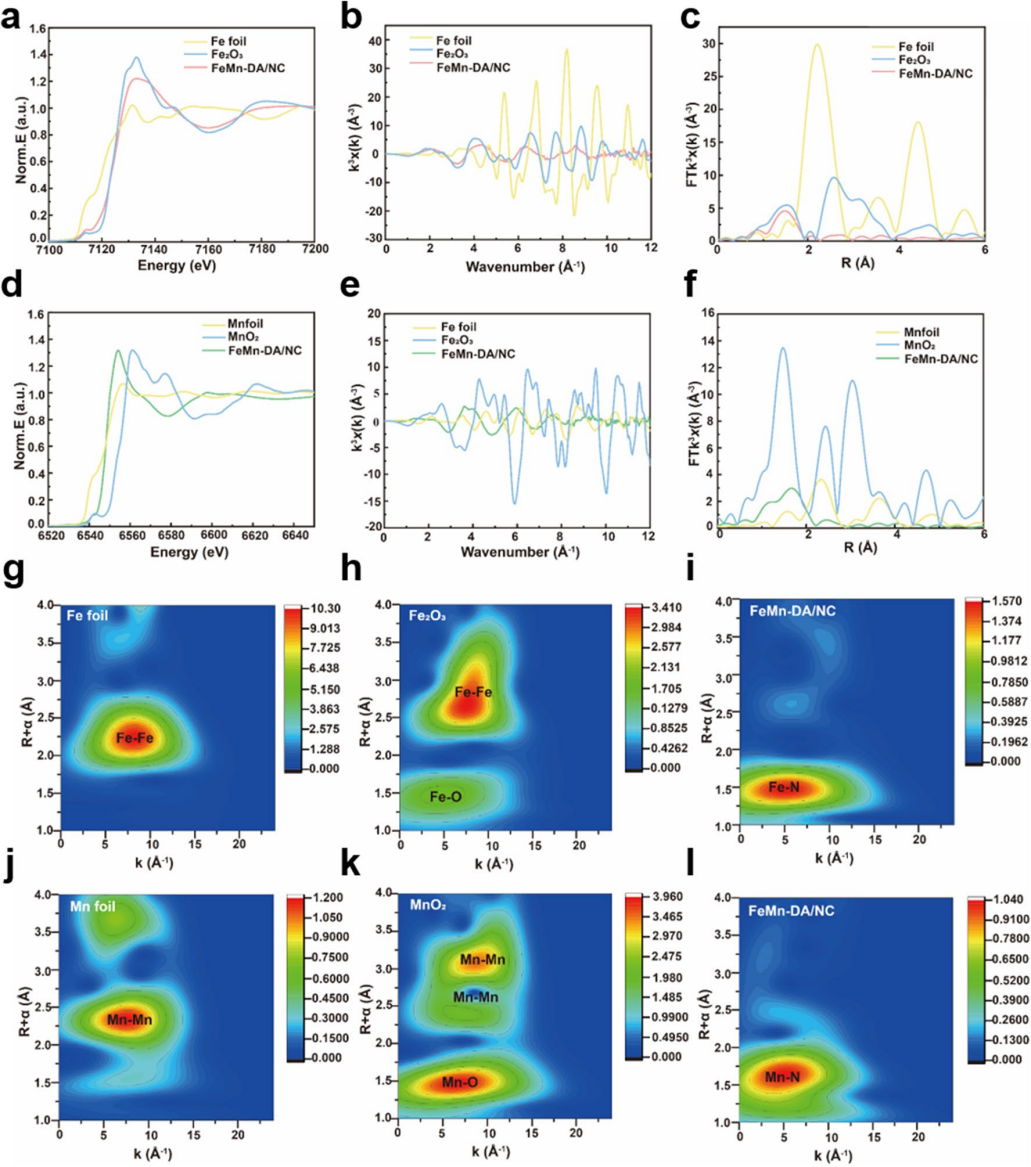
**a** XANES spectra of Fe K-edge of FeMn-DA/NC and reference samples. **b** EXAFS fitting result of Fe in FeMn-DA/NC at K space. **c** Fourier transform (FT) at Fe K-edge of FeMn-DA/NC and reference samples. **d** XANES spectra of Mn K-edge of FeMn-DA/NC and reference samples. **e** EXAFS fitting result of Fe in FeMn-DA/NC at K space.** f** FT at Mn K-edge of FeMn-DA/NC and reference samples. Wavelet transform of **g** Fe foil, **h** Fe_2_O_3_, **i** Fe in FeMn-DA/NC, **j** Mn foil, **k** MnO_2_, and **l** Mn in FeMn-DA/NC

In order to further characterize the Fe and Mn atoms separated by single atoms in FeMn-DA/NC, X-ray absorption near edge structure (XANES) measurements were performed. Firstly, the absorption edge position (~ 7114 eV) of Fe in FeMn-DA/NC between the Fe foil and Fe_2_O_3_, indicating that the valence state of Fe is between 0 and + 3 (Fig. 2a). Subsequently, the extended X-ray absorption Fine structure (EXAFS) and EXAFS fitting curve (Fig. 2b, c) of Fe and coordination parameters (Table S1) were studied, the local coordination environment of Fe site was detected, and the coexistence of four coordination number of N was confirmed [30]. The FT-EXAFS curve of Fe shows that the main peak at 1.47 Å belongs to the Fe–N scattering path, corresponding to the first coordination shell of Fe–N. The absorption edge position of Mn (~ 6540 eV) in FeMn-DA/NC between the Mn foil and MnO_2_, indicating that the valence state of Mn is between 0 and + 4 (Fig. 2d). Subsequently, FT-EXAFS was used to detect local coordination of Mn sites. The FT-EXAFS curve of Mn exhibits a typical Mn-N scattering path at 1.66 Å (Fig. 2e, f). The EXAFS results of Mn and the EXAFS fitting curve (Fig. 2e, f) and coordination parameters (Table S1) show that the Mn–N structure is most likely Mn-N_4_ in FeMn-DA/NC. In addition, wavelet transform (WT) analysis was performed on the EXAFS oscillations at the Fe K edge to distinguish backscattered atoms. The maximum scattering of Fe–Fe in Fe foil is approximately 8.22 Å^–1^ (Fig. 2g). The maximum scattering intensity of FeMn DA/NC is approximately 5.1 Å^–1^, which belongs to Fe–N scattering. The maximum value of Fe–Fe unresponsive intensity (Fig. 2h, i). Similarly, in Mn foil and MnO_2_, the maximum value of WT was observed at Mn–Mn (7.4 Å^–1^) and Mn–O (6.7 Å^–1^) (Fig. 2j, k). FeMn-DA/NC showed a strong response to Mn–Mn deficiency (Fig. 2l). In addition, FeMn-DA/NC exhibits maximum strength at approximately 4.8 Å^–1^, indicating a Mn–N structure. The XANES results indicate that all Fe and Mn are atomic dispersed without any aggregation, which is consistent with ac-HAADF-STEM. Through the above characterization, we confirmed the successful preparation of FeMn-DA/NC, which can be further applied to subsequent experiments.

### Cytotoxicity of DAN

FeMn-DA/NC has higher positive potential (+ 33.1 mV), and were modified with PEG via electrostatic interactions after vigorous stirring and incubation (denoted as DAN (+ 16.7 mV)) to refine the stability, dispersibility and electropositivity, enabling in vitro and in vivo studies (Figs. S8 and S9). The cytotoxicity of DAN was determined by CCK-8 assay in HCE-2 and conjunctival epithelial cells (CCL-20.2). Cells were treated for 24 h at various DAN concentrations ranging from 0.98 to 31.25 μg mL^−1^. As shown in Fig. S10, the viability of cells was maintained at a high level (~ 80%) at investigated DAN concentrations. This indicates DAN exhibit lower cytotoxicity and excellent biocompatibility.

### Effect of DAN to Scavenge ROS Production under Hyperosmolarity

**Fig. 3:**
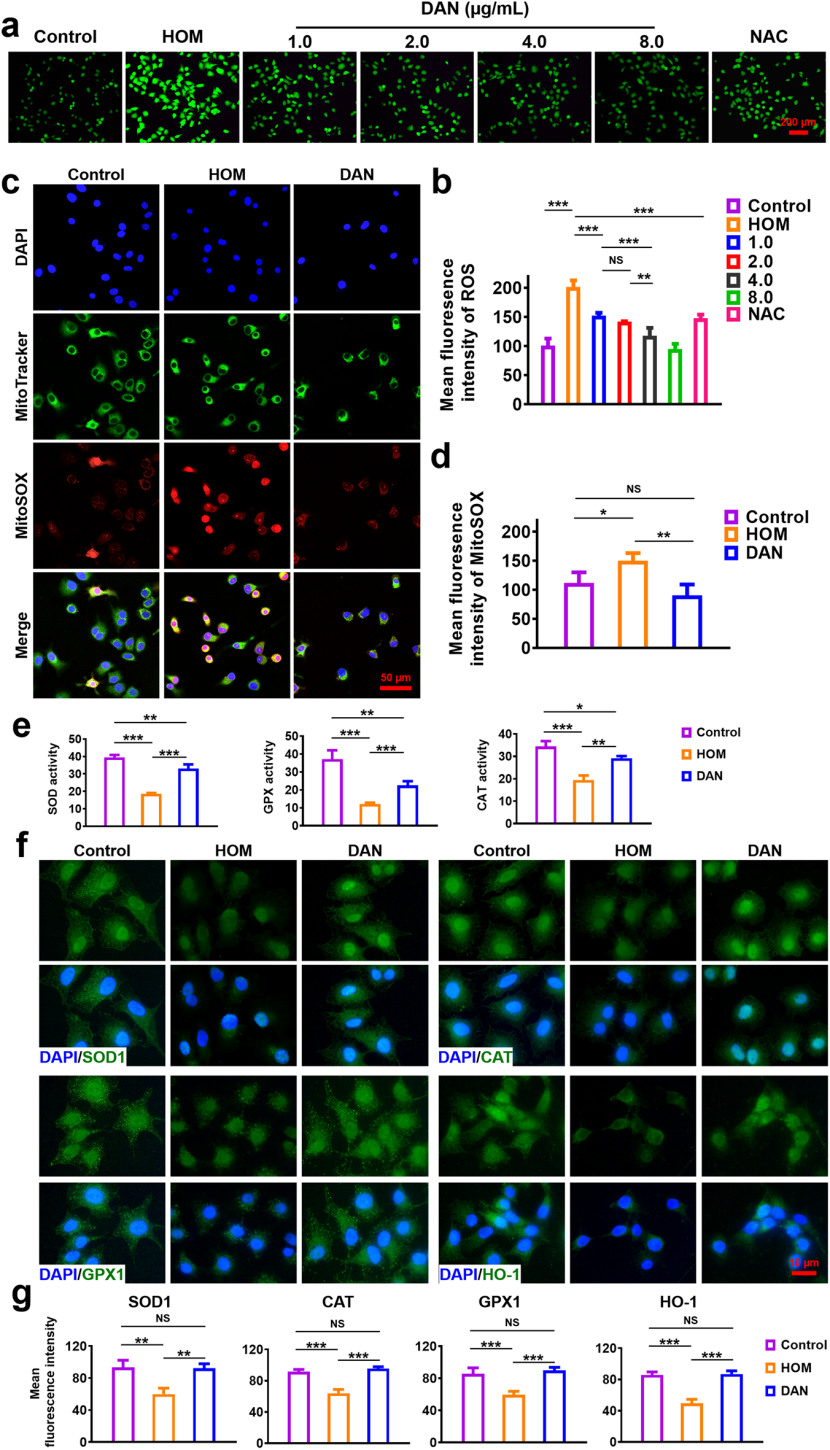
In vitro antioxidant effect of DAN. HCE-2 cells were pre-treated with different concentration of DAN (1, 2, 4 and 8 μg mL^−1^) or NAC (10 mM) before exposure to hypertonic model (HOM) as described in the methods. **a** Total production of intracellular ROS was measured by DCFH-DA assay and **b** quantitative analysis of fluorescence intensity. *Scale bar*: 200 μm. **c** Mitochondria-specific ROS scavenging activity of DAN (2 μg mL^−1^) and **d** quantitative analysis of fluorescence intensity. *Scale bar*: 50 μm. **e** Enzyme activity analysis of SOD, CAT and GPX in hypertonic-stimulated HCE-2 cells pre-treated with or without DAN (2 μg mL.^−1^). **f** Immunofluorescence and **g** quantitative analysis of SOD1, CAT, GPX1 and HO-1 expression. Nuclei were stained with DAPI. *Scale bar*: 10 μm. Data are presented as mean ± SD. *p < 0.05, **p < 0.01, ***p < 0.001 and NS *p* > 0.05

Oxidative stress contributes to the pathogenesis of DED. Hyperosmolarity of the tear film induces elevated ROS, initiating inflammatory cascade in DED. Effective elimination of excess ROS can prevent inflammation and break the dry eye vicious cycle [15]. To determine the ROS-scavenging effect of DAN in vitro*,* ROS production was quantified in HCE-2 cells using a DCFH-DA probe under hypertonic condition (HOM, 500 mOsM), selected to induce ROS overproduction and inflammation (Fig. S11). As shown in Fig. 3a, there is a significant decrease in the DCF fluorescence in DAN groups compared to the hypertonic group, which demonstrated the overexpressed ROS in HCE-2 cells induced by hypertonic stress can be effectively inhibited by DAN. The ROS scavenging effect of DAN at 1 and 2 μg mL^−1^ as effectively as NAC treatment, a well-recognized antioxidant. The quantitative analysis of the fluorescence intensity shown in Fig. 3b was well-consistent with the fluorescent images. The results suggested that DAN can act as an excellent intracellular ROS scavenger.

To further verify mitochondrial ROS-scavenging efficacy of DAN, mitochondrial ROS levels in HCE-2 cells were analyzed via CLSM across three experimental groups. Elevated mitochondrial ROS, evidenced by intense red fluorescence in the mitochondria, was observed in the hypertonic group. This fluorescence was notably reduced following DAN treatment (Fig. 3c, d). Additionally, significant ROS signals were detected in the nuclei of cells exposed to hypertonic conditions, suggesting potential nuclear DNA damage [9]. These observations were consistent with DCFH-DA assay results, confirming the potent antioxidant capabilities of DAN in mitigating hyperosmolarity-induced ROS production in HCE-2 cells.

Oxidative stress arises from an imbalance between ROS and endogenous antioxidant defenses, leading to elevated toxic ROS and oxygen free radicals that cause cell damage [8]. Under normal physiological conditions, intracellular antioxidant enzymes include superoxide dismutase (SOD), glutathione peroxidase (GPX) and catalase (CAT) can maintain cellular redox balance, SOD is responsible for disputing superoxide anions into hydrogen peroxide, while CAT and GPX reduce hydrogen peroxide, thereby preventing the generation of highly toxic hydroxyl radicals leading to apoptosis and inflammation [41]. We assessed the activities of antioxidant enzymes SOD, CAT and GPX across various treatment groups. Compared to the hypertonic group, DAN treated groups exhibited a significant increase in SOD, CAT and GPX activities (Fig. 3e).

Subsequently, we evaluated the expression levels of SOD1, CAT, GPX1 and HO-1 via immunofluorescence staining (Fig. 3f). Heme Oxygenase-1 (HO-1), a key enzyme in heme catabolism, modulates cellular redox homeostasis and has established antioxidant roles in DED [42]. Consistent with enzyme activity data, fluorescence intensities of SOD1, CAT, GPX1, and HO-1 were markedly higher in the DAN group compared to the hyperosmotic group (Fig. 3g), indicating that DAN effectively counteract hyperosmolarity-induced reductions in antioxidant enzyme activity.

### In vitro Cell-protective Activities of DAN

**Fig. 4:**
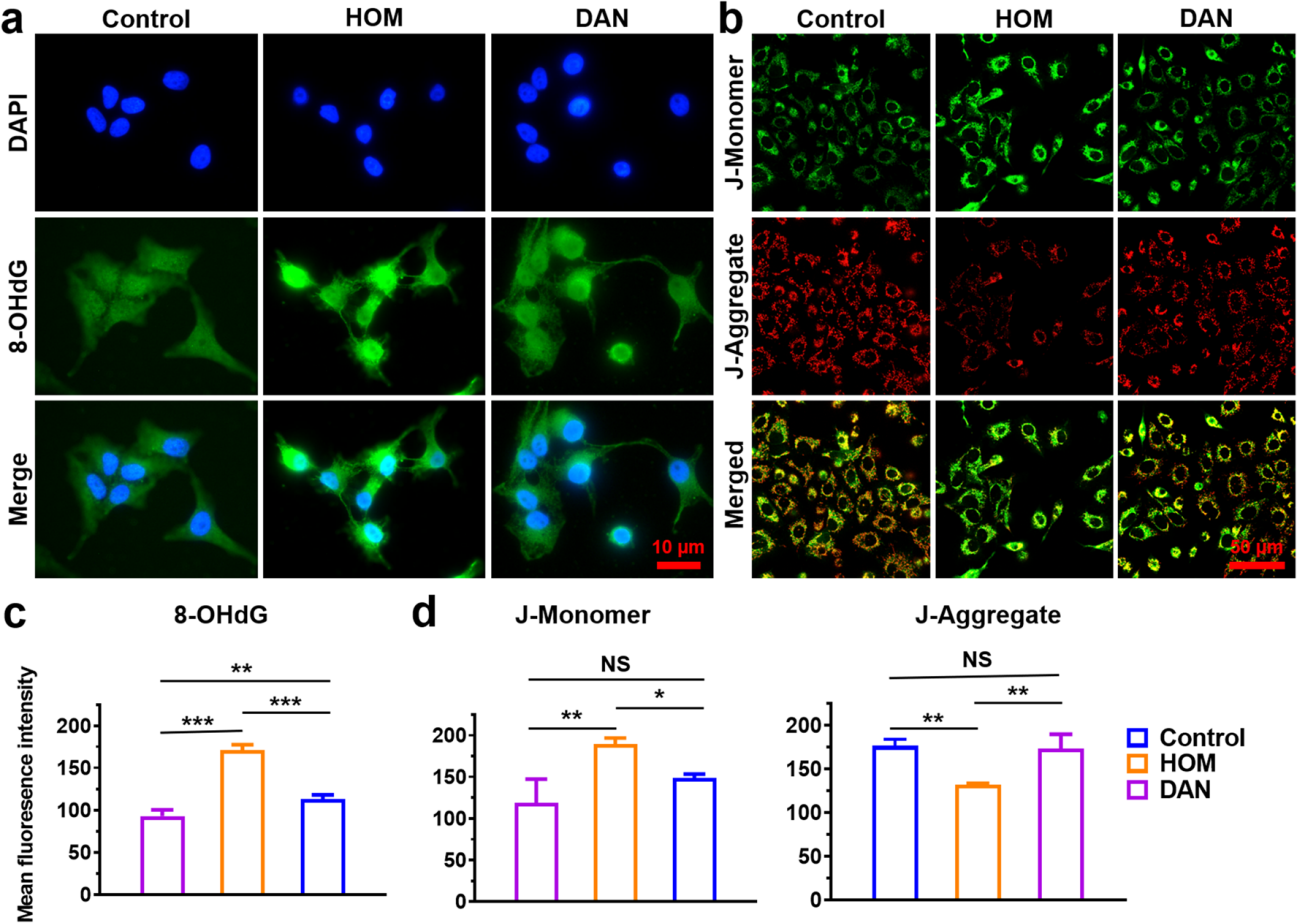
In vitro anti-apoptotic effect of DAN. **a** Immunofluorescent staining of 8-OHdG under hypertonic model or pre-treated with DAN (2 μg mL^−1^) in HCE-2 cells. *Scale bar*: 10 μm. **b** DAN (2 μg mL^−1^) treatment restores mitochondrial membrane potential impairment caused by hypertonicity. *Scale bar*: 50 μm. Quantitative analysis of **c** 8-OHdG and **d** JC-1(J-Monomer and J-Aggregate) fluorescent intensity according to the fluorescence results. Data are presented as mean ± SD. *p < 0.05, **p < 0.01, ***p < 0.001 and NS* p* > 0.05

We confirmed the cytoprotective effect of DAN on hypertonic-induced HCE-2 cell injury using a CCK-8 assay. Exposure to hypertonic medium (500 mOsM) for 24 h significantly reduced cell viability to an average of 50%. However, DAN at concentrations of 2, 4, and 8 μg mL^−1^ improved cell viability to 62%, 60%, and 55%, respectively (Fig. S12). A concentration of 2 μg mL^−1^, yielding the highest cell viability, was selected for subsequent cell experiments. Additionally, fluorescence of 8-OHdG, an established marker for oxidative DNA damage, was markedly elevated in hypertonic-exposed HCE-2 cells but was significantly suppressed by DAN treatment (Fig. 4a, c).

Oxidative stress can compromise mitochondrial integrity and function [43]. A decline in the mitochondrial membrane potential, indicative of early apoptosis, can be detected by the JC-1 dye transition from red to green fluorescence. To assess ROS-induced damage to the inner mitochondrial membrane and evaluate the protective effects of DAN on mitochondrial membrane potential, we used JC-1 fluorescent probing across experimental groups. As shown in Fig. 4b, DAN treatment notably augmented red fluorescence and diminished green fluorescence relative to the hypertonic group, implying effective maintenance of membrane potential under oxidative stress. No significant difference in red and green fluorescence intensities was observed between the DAN and negative control groups (Fig. 4d), indicating DAN’s robust cytoprotective efficacy against hypertonic-induced oxidative damage.

### Inhibiting NLRP3 Inflammasome Activation by Attenuating Oxidative Stress in vitro

**Fig. 5:**
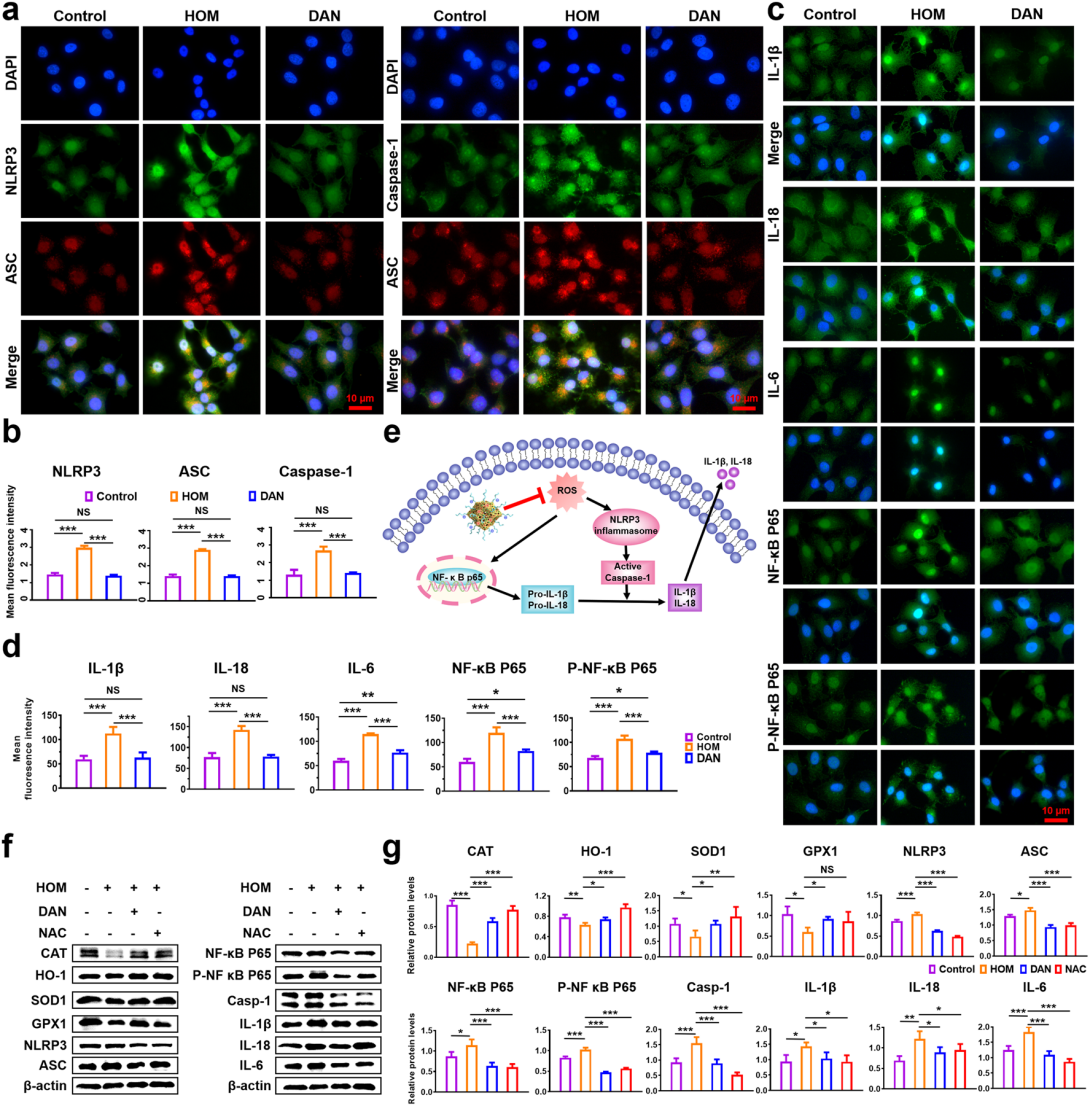
In vitro antioxidant and anti-inflammatory mechanisms of DAN. **a** Double immunofluorescent staining and **b** quantitative analysis of fluorescent intensity results showed the fluorescence intensity of NLRP3, ASC and Caspase-1 were significantly increased in HCE-2 cells under hypertonic conditions, which were completely suppressed by DAN (2 μg mL^−1^). **c** Immunofluorescence and **d** quantitative analysis of fluorescent intensity results showed that DAN inhibited the expressions of IL-1β, IL-18, IL-6, NF-κB P65 and P-NF-κB P65. *Scale bar*: 10 μm. **e** Schematic representation of the inhibitory mechanism of intracellular signaling pathways and the targets of DAN. **f** The protein expression of SOD1, CAT, GPX1, HO-1, NLRP3, ASC, Caspase-1, IL-1β, IL-18, IL-6, NF-κB P65 and P-NF-κB P65 in the cell lysates derived from HCE-2 cells were analyzed by western blotting and **g** densitometry analyses of the western blotting results. Data are presented as mean ± SD. *p < 0.05, **p < 0.01, ***p < 0.001 and NS *p* > 0.05

Excessive ROS can activate the NLRP3 inflammasome, triggering a cascade that promotes IL-1β and IL-18 secretion and subsequent ocular surface inflammation (Fig. 5e). To investigate DAN’s potential in inhibiting NLRP3 inflammasome activation, given their robust ROS-scavenging and multi-enzyme-like activities, HCE-2 cells were pre-incubated with or without 2 μg mL^−1^ DAN prior to 24 h exposure to hypertonic medium (500 mOsM). Immunofluorescent data showed that NLRP3/ASC/Caspase-1 inflammasomes were activated in HCE-2 cells exposed to hyperosmotic medium, which was completely reversed by DAN (Fig. 5a, b). Given that extracellular secretion of caspase-1-cleaved IL-1β and IL-18 serves as an indicator of NLRP3 inflammasome activation, we assessed the expression levels of these cytokines in each experimental group using immunofluorescence. DAN treatment dramatically reduced the fluorescence intensities of hypertonicity-induced IL-1β and IL-18 signals (Fig. 5c, d). Meanwhile, ROS can activate the NF-κB signaling pathway, upregulating pro-IL-1β and pro-IL-18 expression. Immunofluorescent data showed that DAN treatment attenuated the expressions of NF-κB P65 and P-NF-κB P65 in hypertonicity-induced HCE-2 cells (Fig. 5c, d). In addition, the expression of IL-6, an important proinflammatory cytokine, was also examined (Fig. 5c, d).

We further evaluated proteins involved in the ROS-NLRP3-IL-1β inflammatory axis in hyperosmotic stress-induced HCE-2 cells using western blotting. Consistent with immunofluorescence findings, levels of NLRP3, ASC, Caspase-1, IL-1β, IL-18, IL-6, NF-κB P65 and P-NF-κB P65 were significantly elevated in the hyperosmotic group but attenuated upon DAN treatment (Fig. 5f, g). Similarly, DAN restored the reduced expression of hypertonic-induced antioxidant proteins CAT, HO-1, GPX1, and SOD1(Fig. 5f, g). These results conclusively demonstrated that DAN inhibit NLRP3 inflammasome activation by scavenging excess ROS.

### In vivo Therapeutic Effects on DED

**Fig. 6:**
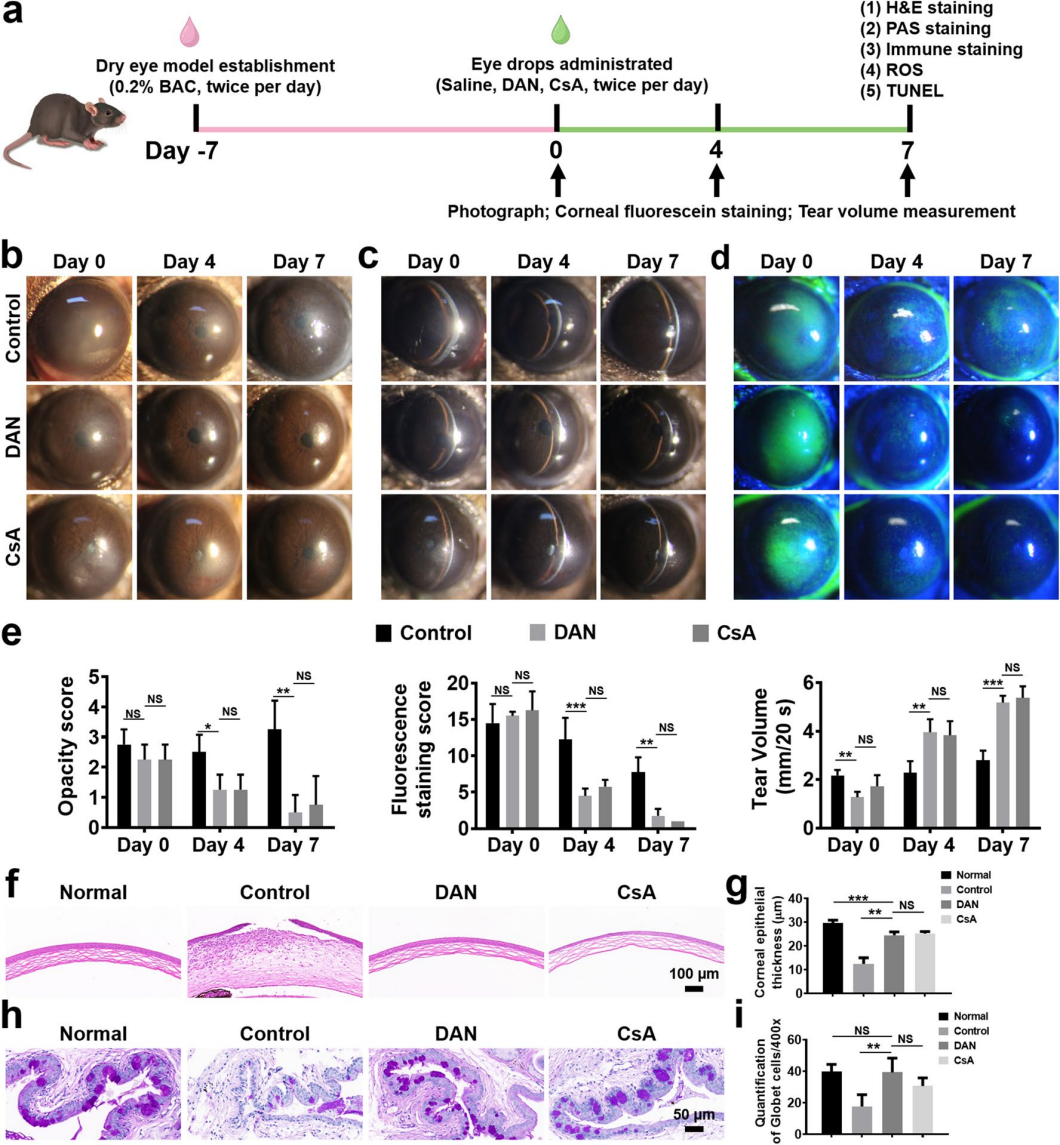
Therapeutic efficacy of DAN on BAC-induced mice model of DED. **a** Timeline of the establishment of dry eye mice model and drug administration. After instillation with 5 μL 0.2% w/v benzalkonium chloride twice per day for 7 days (defined as day 0), the mice were treated with 5 μL of 0.9% w/v saline, DAN (100 μg mL^−1^), and 0.05% CsA twice per day, respectively. The therapeutic effects were evaluated and recorded during the therapy process. **b** Optical, **c** slit-lamp and **d** corneal fluorescein-stained micrographs of mice eyes under different treatments. **e** Opacity scores, fluorescein staining scores and tear volume of the DED mice treated with different topical eye drop solutions. **f** Representative H&E staining images of the cornea and **g** quantitative analysis of the corneal epithelial thickness. *Scale bar*: 100 μm. **h** Representative conjunctival PAS staining images and **i** numbers of goblet cells in each field. Three stained sections of each mouse conjunctiva were counted for statistical analysis. *Scale bar*: 50 μm. Data are presented as mean ± SD. *p < 0.05, **p < 0.01, ***p < 0.001 and NS* p* > 0.05

To investigate the in vivo therapeutic efficacy of DAN for DED, a mouse dry eye model was established using topical 0.2% benzalkonium chloride (BAC), a commonly used agent for inducing DED with acute corneal epithelial defects and excessive ROS production [44]. 0.05% CsA, a clinically approved DED treatment, served as a positive control. Post 7-day treatment with 0.2% BAC eye drops twice daily, mice received DAN (100 μg mL^−1^) or CsA eye drops (Fig. 6a). Corneal pathology was assessed via slit-lamp microscopy and scored based on established criteria at days 0, 4, and 7 post-treatments [36, 37].

As depicted in Fig. 6b–e, corneal edema, opacity, epithelial defect and tear secretion deficiency were observed in all groups post-7-day BAC induction, confirming successful DED model establishment. Fluorescein staining revealed that minimal staining in the DAN and CsA groups at day 4. By day 7, plaque staining was evident in the control group, while the DAN and CsA groups exhibited negligible micropunctate staining (Figs. 6d and S13). Compared to controls, DAN and CsA treatments resulted in reduced corneal opacity and fluorescein staining scores on day 4 and day 7 (Fig. 6e). Average tear volumes, an important clinical index in the diagnosis of DED symptoms, were 3.02 ± 0.60 mm, 5.18 ± 0.29 mm, and 5.38 ± 0.48 mm for the control, DAN, and CsA groups at day 7, respectively (Fig. 6e).

Histological analysis (Fig. 6f) revealed defective, thin corneal epithelium and stromal inflammatory infiltration in the control group at day 7. In contrast, DAN and CsA preserved corneal integrity and reduced inflammation, approximating normal morphology (Fig. 6g).

PAS staining was used to label conjunctival goblet cells (Fig. 6h) which secrete mucus to maintain ocular surface homeostasis and prevent dry eye syndrome and are susceptible to the oxidative ocular environment [45]. The control group exhibited significant goblet cell loss and atrophy, potentially reducing mucin secretion. DAN treatment significantly restored the number of goblet cells (*p* < 0.01, Fig. 6i), comparable to 0.05% CsA treatment. Overall, DAN effectively ameliorated corneal and conjunctival abnormalities and restored tear secretion in DED models.

### In vivo Underlying Mechanisms

**Fig. 7:**
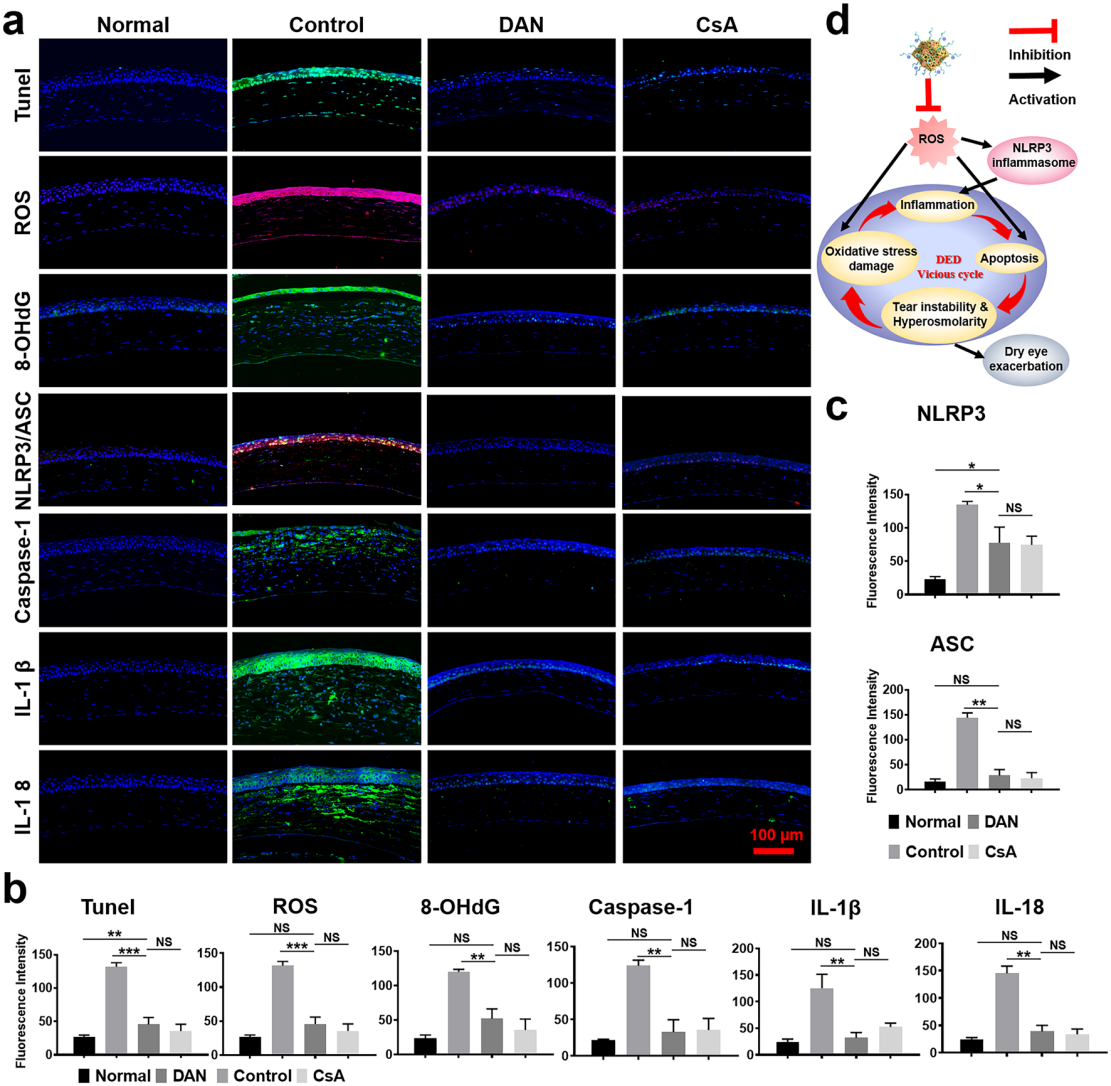
In vivo antioxidant, anti-apoptotic, anti-inflammatory effect of DAN. **a** Evaluations and **b, c** quantitative analysis of fluorescent intensity of ROS, apoptosis (TUNEL), 8-OHdG, NLRP3, ASC, Caspase-1, IL-1β and IL-18 expression were determined by immunofluorescence staining on the corneal epithelium in the mice eyes after topical administration of saline, DAN, CsA for DED. *Scale bar*: 100 μm. **d** Schematic diagram of the in vivo therapeutic mechanism of DED using DAN to suppress inflammation and break vicious cycle by scavenging excess ROS. Data are presented as mean ± SD. **p < 0.01, ***p < 0.001 and NS* p* > 0.05

The ROS-NLRP3-IL-1β signaling axis is implicated as a key inhibitor of ocular inflammation in DED [46]. To delineate DAN’s therapeutic targets in DED (Fig. 7d), we assessed antioxidant and anti-inflammatory, and anti-apoptotic markers in corneal tissue via immunofluorescence, including ROS, NLRP3/ASC, IL-1 β, IL-18, 8-OHdG, and TUNEL assays (Fig. 7a–c). DHE staining confirmed that DAN’s in vivo efficacy is linked to ROS scavenging, as evidenced by reduced ROS levels in the DAN and CsA groups compared to controls (Fig. 7a).

Given the pivotal role of ocular surface epithelial cell apoptosis in DED progression, we further examined DAN’s anti-apoptotic effects using TUNEL assays. Few TUNEL-positive cells were observed in normal corneas, while BAC-induced controls exhibited numerous TUNEL-positive cells. DAN treatment significantly attenuated corneal epithelial apoptosis, confirming its potent in vivo therapeutic efficacy (Fig. 7a).

8-OHdG serves as a biomarker for DNA oxidative damage. Both DAN and CsA treatments resulted in reduced 8-OHdG expression in comparison to controls (Fig. 7a, b). ROS are the main mediators of NLRP3 inflammasome activation. It has been demonstrated that excessive ROS can activate NLRP3 inflammasome, leading to its downstream factor, ASC, Caspase-1, IL-1β, and IL-18 are elevated in DED. Immunofluorescence data revealed elevated expression of NLRP3, ASC, Caspase-1, IL-1β, and IL-18 in the DED group, which was substantially mitigated by DAN and CsA (Figs. 7a–c and S14). Notably, DAN demonstrated superior IL-1β suppression compared to CsA, possibly due to its enhanced ROS removal capability. Taken together, these findings suggested that DAN protect against DNA oxidative damage and blocks NLRP3 inflammasomes activation by attenuating excessive ROS.

### In vivo Safety Assessment

Following twice daily instilment of DAN (100 μg mL^−1^) for 7 days, ocular tissues (cornea, conjunctiva, iris, lens, retina) and major organs were harvested to evaluate the toxicity of DAN. H&E staining revealed no discernible pathologic alterations in DAN-treated mice compared to normal group (Fig. S15). In addition, no gross or histopathological abnormalities were observed in heart, liver, spleen, lung, and kidney tissues. In summary, DAN demonstrated excellent ocular biocompatibility and negligible systemic or corneal toxicity in vivo.

## Conclusion

In conclusion, a novel antioxidative and dual-atom nanozyme was successfully prepared by embedding Fe and Mn bimetallic single-atoms in N-doped carbon material and modifying it with a hydrophilic polymer. Our fabricated DAN suppressed the hypertonicity-induced inflammatory responses in HCE-2 cells by inhibiting ROS/NLRP3 pathways. Furthermore, DAN could both reduce intracellular oxidative stress and improve the therapeutic effects on inhibiting corneal epithelial injury, protecting goblet cells, and promoting tear secretion in the dry eye animal models. Our studies revealed the great potential of the ROS-scavenge nanozyme in the DED treatment due to its strong biological function, good biocompatibility, powerful ability to scavenge excessive ROS and alleviate inflammation which indicated a promising application for ocular surface disease therapeutics. However, non-organic nanozymes, such as SAzyme, has high activity like natural enzyme. It is inevitable that the biosafety and stability have always been the main problems. Therefore, the subsequent research will improve the biological safety and stability of the non-organic nanozymes.

## Supplementary Information

Below is the link to the electronic supplementary material.

Supplementary file1 (PDF 1272 KB)
