# Supplementary material for: Dual-Atom Nanozyme Eye Drops Attenuate Inflammation and Break the Vicious Cycle in Dry Eye Disease
Source: Nanomicro Lett. 2024 Feb 19;16:120. doi: 10.1007/s40820-024-01322-7 (PMC10876514; doi:10.1007/s40820-024-01322-7)
Supplement: Supplementary file 1 — Supplementary file1 (PDF 1272 KB) [file 40820_2024_1322_MOESM1_ESM.pdf]

Supporting Information for

Dual-Atom Nanozyme Eye Drops Attenuate Inflammation and Break the Vicious Cycle in Dry Eye Disease

Dandan Chu[1], Mengyang Zhao[1,*], Shisong Rong[2,*], Wonho Jhe[3], Xiaolu Cai[4], Yi Xiao[3], Wei Zhang[1], Xingchen Geng[1], Zhanrong Li[1,*], Xingcai Zhang[3,*], Jingguo Li[1,*]

[1] Henan Eye Hospital, Henan Provincial People's Hospital, People's Hospital of Zhengzhou University, Zhengzhou, 450003, P. R. China

[2] Department of Ophthalmology, Mass Eye and Ear, Mass General Brigham, Harvard Medical School, Boston, MA, 02114, USA

[3] School of Engineering and Applied Sciences, Harvard University, Cambridge, MA, 02138, USA

[4] College of Chemistry, Zhengzhou University, Zhengzhou, Henan, 450001, P. R. China

*Corresponding authors. E-mail: zhaomyscnu@163.com (Mengyang Zhao); shisong_rong@meei.harvard.edu (Shisong Rong); lizhanrong@zzu.edu.cn (Zhanrong Li); lijingguo@zzu.edu.cn (Jingguo Li); xingcai@seas.harvard.edu and zhangxingcai@wteao.com (Xincai Zhang)

S1 Experimental and Methods

S1.1 Materials

The 2-methylimidazole, zinc nitrate hexahydrate ($Zn(NO_3)_2 \cdot 6H_2O$), anhydrous manganese chloride ($MnCl_2$), ferric acetylacetonate ($Fe(acac)_3$), tetraethyl orthosilicate (TEOS), sodium hydroxide (NaOH), hexadecyltrimethylammonium bromide (CTAB), methanol, hydrofluoric acid (HF), hydrochloric acid (HCl), ethanol, PEG_{1000} were purchased from Shanghai Aladdin Biochemical Technology Co., Ltd. (Shanghai, China). All reagents were used as received, without further purification.

Cell counting Kit-8 (CCK-8) was purchased from Dojindo (Japan). 2′,7′-dichlorofluorescein diacetate (DCFH-DA), N-acetyl-L-cysteine (NAC), Fluoroshield™ with DAPI and dihydroethidium (DHE) were purchased from Sigma-Aldrich (St Louis, MO, USA). Sodium chloride (NaCl) was purchased from Tianjin Hengxing Chemical Preparation Co., Ltd. MitoTracker™ Green FM and MitoSOX™ Red mitochondrial superoxide tracker was purchased from Invitrogen. DAPI solution, Superoxide Dismutase (SOD) (BC0170), Glutathione Peroxidase (GSH-Px/GPX) (BC1190) and Catalase (CAT) (BC0205) activity assay kits were purchased from Solarbio Science & Technology Co., Ltd. (Beijing, China). Mitochondrial membrane potential assay kit with JC-1 (C2006) was purchased from Beyotime Biotechnology (Shanghai, China). SOD1 (bs-10216R), GPX1 (bs-3882R), CAT (bs-6874R), HO-1 (bs-

2075R), NLRP3 (bs-6655R), Caspase-1 (bs-0169R), IL-1β (bs-0812R), 8-OHdG (bs-1278R) antibodies were purchased from Bioss. ASC (sc-514414) antibody was purchased from Santa Cruz Biotechnology. NF-κB P65 (AF5006), P-NF-κB P65 (AF2006), IL-18 (DF6252), IL-6 (DF6087) antibodies were purchased from Affinity. Secondary antibody FITC-labeled goat anti-rabbit IgG H&L (A0562) and Cy3-labeled goat anti-mouse IgG (H+L) (A0521) were purchased from Beyotime. Goat anti-rabbit-IgG-HRP (abs20040) and goat anti-mouse-IgG-HRP (abs20039) were purchased from Absin. Benzalkonium chloride was purchased from Hubei Gedian Humanwell Pharmaceutical Excipients Co., Ltd. TUNEL staining kit was purchased from Roche Co., Ltd., Germany.

S1.2 Characterization

Computed tomography (CT) X-ray diffraction (Empyrean XRD) measurements were carried out using an X-ray diffractometer with a Mn Kα radiation source (45 kV, 40 mA). X-ray photoelectron spectroscopy (XPS) spectra were obtained using an X-ray photoelectron spectrometer (AXIS Supra, Shimadzu/Kratos, England) equipped with an Al Kα radiation source. Calibration was performed with adsorption C 1s 284.8 eV as the standard. The appearance of the samples was observed using a field emission scanning electron microscope (FESEM, Auriga FIB, Zeiss, Germany). An FEI TalosF200S transmission electron microscope (TEM; Czech Republic) characterized the morphology and selected area electron diffraction (SAED). HAADF-STEM and high-resolution STEM (HR-STEM) were performed on a JEOL ARM-300F with spherical aberration correction operated at 300 kV. Metal element contents were confirmed by inductively coupled plasma mass spectrometry (ICP-MS; NWR-213). The scanning electron microscope (SEM) images were obtained by a scanning electron microscope (ZEISS Sigma 500, USA).

S1.3 XAFS measurements and simulations

Data reduction, data analysis, and EXAFS fitting were performed and analyzed with the Athena and Artemis programs of the Demeter data analysis packages that utilizes the FEFF6 program to fit the EXAFS data [S1, S2]. The energy calibration of the sample was conducted through standard and Fe foil and Mn foil, which as a reference was simultaneously measured. A linear function was subtracted from the pre-edge region, then the edge jump was normalized using Athena software. The $\chi(k)$ data were isolated by subtracting a smooth, third-order polynomial approximating the absorption background of an isolated atom. The $k3$-weighted $\chi(k)$ data were Fourier transformed after applying a HanFeng window function ($\Delta k = 1.0$). For EXAFS modeling, the global amplitude EXAFS (CN, R, σ^2 and ΔE_0) were obtained by nonlinear fitting, with least-squares refinement, of the EXAFS equation to the Fourier-transformed data in R-space, using Artemis software, EXAFS of the Fe foil and Mn foil are fitted and the obtained amplitude reduction factor S_0^2 value (0.710 and 0.816) was set in the EXAFS analysis to determine the coordination numbers (CNs) in the Fe–O, Fe–Fe and Fe–Mn scattering path in sample.

S1.4 Cell culture

The human corneal epithelial cell line HCE-2 and the conjunctival epithelial cell line CCL-20.2 were purchased from American Type Culture Collection (Manassas, VA, USA). Cells were cultured in Dulbecco's modified Eagle's medium/nutrient mixture F-12 (DMEM/F12, Solarbio, Beijing, China) supplemented with 10% fetal bovine serum (FBS, Gibco), 100 U mL^{-1} penicillin, and 100 µg/mL streptomycin (Solarbio, Beijing, China) at 37 °C in a 5% CO_2 incubator.

S1.5 *In vitro* cytocompatibility

The cytotoxicity test of DAN was investigated in HCE-2 and CCL-20.2 cells using a Cell Counting Kit 8 assay. HCE-2 and CCL-20.2 cells were seeded onto 96-well plates at a density of 8×10^3 cells per well and 10×10^3 cells per well, respectively, and cultured in 5% CO_2 at 37 °C for 24 h. Subsequently, the cells were treated with various concentrations of DAN (0, 0.98, 1.95, 3.9, 7.8, 15.6, 31.25 µg/mL) diluted with DMEM/F12 medium respectively for another 24 h. Then the medium was removed and a fresh medium (100 µL) containing 10% CCK-8 solution was added to each well for an additional 4 h at 37 °C. The optical density (OD values) was measured at 450 nm using an Enzyme Labeler (PerkinElmer EnVision, England). Cells cultured with medium alone were used as control.

S1.6 *In vitro* hypertonic model

HCE-2 cells were seeded in 96-well plates at a density of 8×10^3 cells per well and incubated overnight at 37 °C. Then the cells were switched to a serum-free DMEM/F12 medium for 6 h before treatment. Next the cells were treated for 24 h in serum-free medium with iso- and hyper- osmolarity (310, 400, 450 and 500 mOsM), which was achieved by adding 0, 50, 70, or 90 mM sodium chloride (NaCl). The osmolarity of the culture media was measured by osmometer (STY-1A, Tianjin Tianda Technology Co., Ltd.). Then the medium was removed and a fresh serum-free medium (100 µL) containing 10% CCK-8 solution was added to each well for an additional 4 h at 37 °C. The optical density (OD values) was measured at 450 nm using an Enzyme Labeler. Similarly, Cells were washed twice with PBS and stained for 30 min at 37° C with 10 µM DCFH-DA in the dark. The ROS (DCF) fluorescence intensity was detected by cytation5 imaging reader (Bioteck, America).

S1.7 *In vitro* anti-apoptotic properties

HCE-2 cells were seeded in 96-well plates at a density of 8×10^3 cells per well and incubated overnight at 37 °C. Then the cells were pretreated with serum-free DMEM/F12 medium containing different concentrations of DAN (1, 2, 4, 8 µg/mL) for 6 h. Subsequently, the cells were cultured in serum-free DMEM-F12 containing 90 mM NaCl, which was used to create a hypertonic environment (500 mOsM) for an additional 24 h. The ROS inhibitor NAC (10 mM) was added 1 h before NaCl supplementation as a positive control. Then the medium was removed and a fresh serum-free medium (100 µL) containing 10% CCK-8 solution was added to each well for an additional 4 h at 37 °C. The optical density (OD values) was measured at 450 nm using an Enzyme Labeler (PerkinElmer EnVision, England). Cells cultured with

medium alone were used as control.

S1.8 Safety evaluation

On the 7th day after treatment with DAN, the ocular tissues, including cornea, conjunctiva, iris, lens and retina as well as the organs such as heart, liver, spleen, lung, and kidney were quickly extracted from the mice and made into H&E stained specimens for further study of *in vivo* biological safety.

S2 Supplementary Tables and Figures

Table S1 EXAFS fitting parameters at the Fe and Mn *K*-edge for various samples

<table>
<tr><th>Sample</th><th>Shell</th><th>CN^a</th><th>R (Å)^b</th><th>σ² (Å²)^c</th><th>ΔE₀ (eV)^d</th><th>R factor</th></tr>
<tr><td colspan="7">Fe</td></tr>
<tr><td rowspan="2">Fe-foil</td><td>Fe–Fe</td><td>8*</td><td>2.462 ± 0.017</td><td>0.0049 ± 0.0018</td><td>5.3 ± 2.7</td><td rowspan="2">0.0060</td></tr>
<tr><td>Fe–Fe</td><td>6*</td><td>2.842 ± 0.022</td><td>0.0050 ± 0.0028</td><td>4.6 ± 3.9</td></tr>
<tr><td rowspan="3">Fe2O3</td><td>Fe–O</td><td>6.0 ± 0.5</td><td>1.982 ± 0.001</td><td>0.0134 ± 0.0016</td><td>−1.5 ± 0.4</td><td rowspan="3">0.0024</td></tr>
<tr><td>Fe–Fe</td><td>5.9 ± 0.6</td><td>2.982 ± 0.001</td><td>0.0080 ± 0.0009</td><td>3.4 ± 0.3</td></tr>
<tr><td>Fe–Fe</td><td>2.3 ± 0.5</td><td>3.650 ± 0.001</td><td>0.0008 ± 0.0014</td><td>−9.2 ± 0.6</td></tr>
<tr><td>FeMn-DA/N (Fe)</td><td>Fe–N</td><td>4.3 ± 0.3</td><td>1.966 ± 0.001</td><td>0.0101 ± 0.0015</td><td>−0.8 ± 0.4</td><td>0.0067</td></tr>
<tr><td colspan="7">Mn</td></tr>
<tr><td>Mn-foil</td><td>Mn–Mn</td><td>2*</td><td>2.676 ± 0.001</td><td>0.0064 ± 0.0017</td><td>7.8 ± 0.8</td><td>0.0151</td></tr>
<tr><td rowspan="3">MnO2</td><td>Mn–O</td><td>6.0 ± 0.5</td><td>1.881 ± 0.001</td><td>0.0025 ± 0.0009</td><td>0.4 ± 0.6</td><td rowspan="3">0.0068</td></tr>
<tr><td>Mn–Mn</td><td>3.2 ± 0.9</td><td>2.842 ± 0.001</td><td>0.0053 ± 0.0024</td><td>−9.0 ± 1.2</td></tr>
<tr><td>Mn–Mn</td><td>5.8 ± 1.0</td><td>3.439 ± 0.001</td><td>0.0024±0.0013</td><td>0.1±0.7</td></tr>
<tr><td rowspan="2">FeMn-DA/NC (Mn)</td><td>Mn–N</td><td>1.4 ± 0.2</td><td>1.997 ± 0.001</td><td rowspan="2">0.0050±0.0026</td><td rowspan="2">−4.2±0.9</td><td rowspan="2">0.0099</td></tr>
<tr><td>Mn–N</td><td>2.8 ± 0.4</td><td>2.166 ± 0.001</td></tr>
</table>

[a] *CN*, coordination number; [b] *R*, the distance to the neighboring atom; [c] σ^2, the Mean Square Relative Displacement (MSRD); [d] ΔE_0, inner potential correction; *R* factor indicates the goodness of the fit. $S0^2$ was fixed to 0.710 and 0.816, according to the experimental EXAFS fit of Fe foil and Mn foil by fixing *CN* as the known crystallographic value. * This value was fixed during EXAFS fitting, based on the known structure of Fe and Mn. Fitting range: $3.0 \leq k$ (/Å) ≤ 12.0 and $1.0 \leq R$ (Å) ≤ 3.0 (Fe foil); $3.0 \leq k$ (/Å) ≤ 12.0 and $1.0 \leq R$ (Å) ≤ 3.5 (Fe_2O_3); $3.0 \leq k$ (/Å) ≤ 12.0 and $1.0 \leq R$ (Å ≤ 2.0 (322-Fe); $3.0 \leq k$ (/Å) ≤ 12.0 and $1.9 \leq R$ (Å) ≤ 2.9 (Mn foil); $3.0 \leq k$ (/Å) ≤ 12.0 and $1.0 \leq R$ (Å) $\leq$ 3.5 (MnO_2); $3.0 \leq k$ (/Å) ≤ 11.0 and $1.0 \leq R$ (Å) ≤ 2.3 (322-Mn). A reasonable range of EXAFS fitting parameters: $0.700 < S_0^2 < 1.000$; $CN > 0$; $\sigma^2 > 0$ Å^2; $|\Delta E_0| < 15$ eV; R factor < 0.02

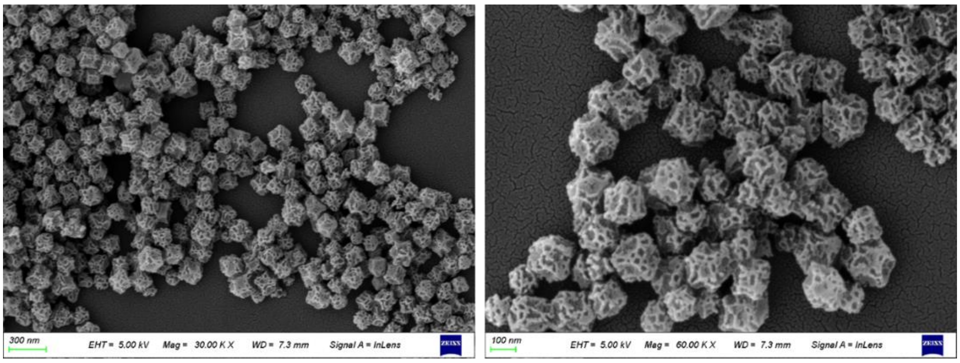

Fig. S1 SEM image of FeMn-DA/NC

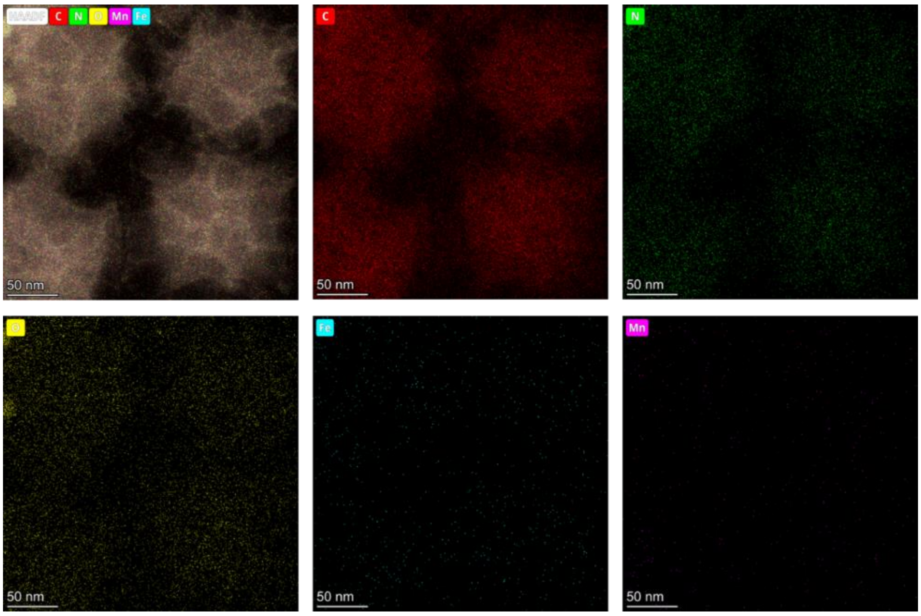

Fig. S2 Corresponding energy dispersive spectroscopy (EDS) mappings of FeMn-DA/NC, where C (red), N (green), O (yellow), Fe (cyan), and Mn (amaranth) were imaged under the STEM mode

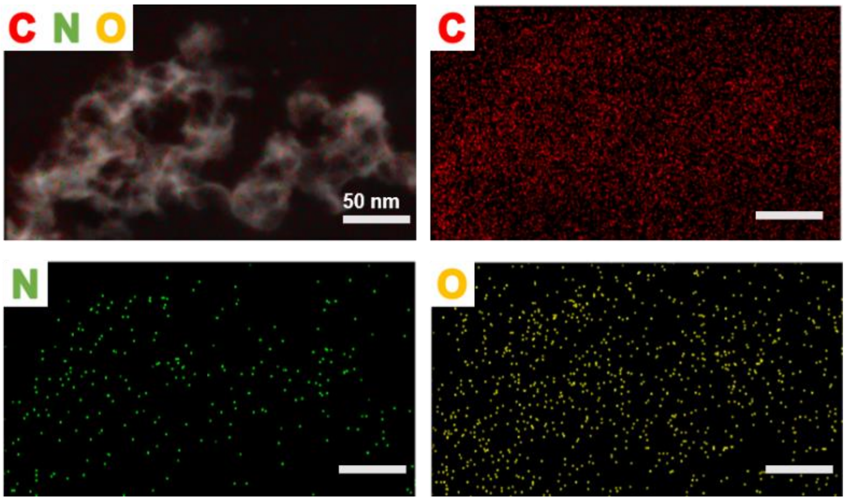

Fig. S3 Corresponding EDS mappings of DA/NC, where C (red), N (green), O (yellow), were imaged under the STEM mode

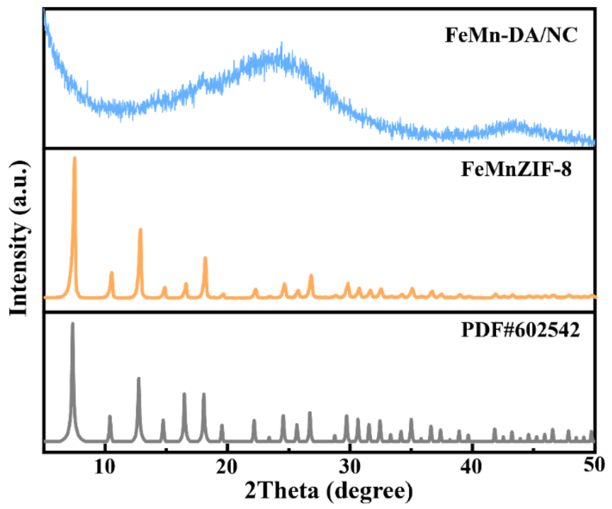

Fig. S4 XRD patterns of FeMn-DA/NC and FeMnZIF-8

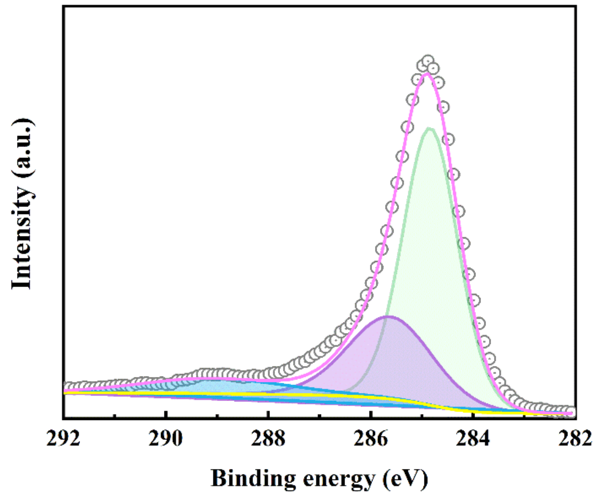

Fig. S5 C 1s XPS spectra of FeMn-DA/NC

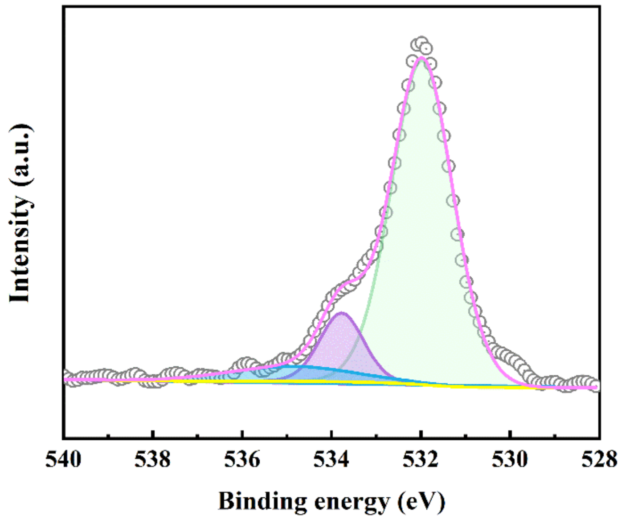

Fig. S6 O 1s XPS spectra of FeMn-DA/NC

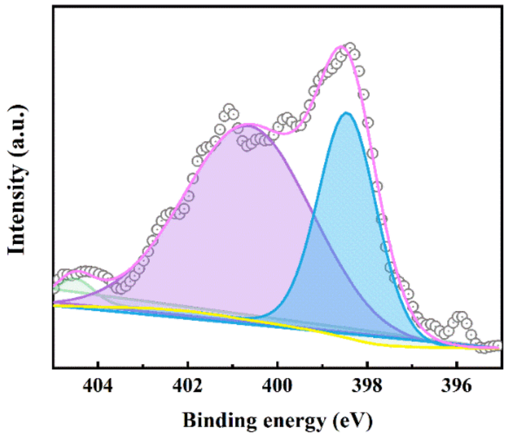

Fig. S7 N 1s XPS spectra of FeMn-DA/NC

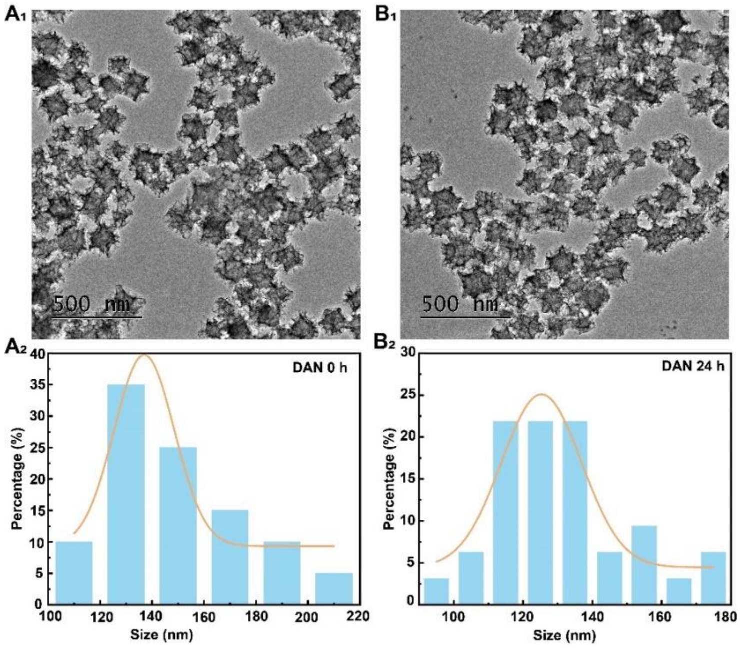

Fig. S8 TEM images (A1, B1) and particle size analysis (A2, B2) of DAN in 24 h

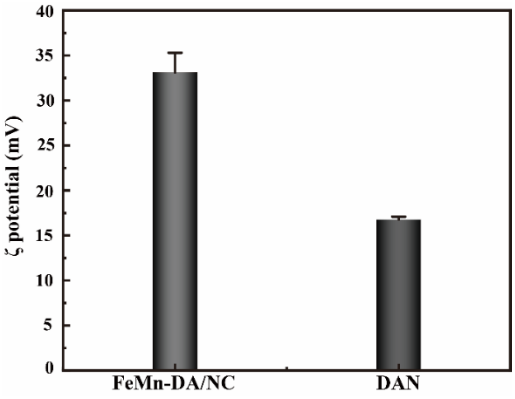

Fig. S9 ζ potential of FeMn-DA/NC and DAN

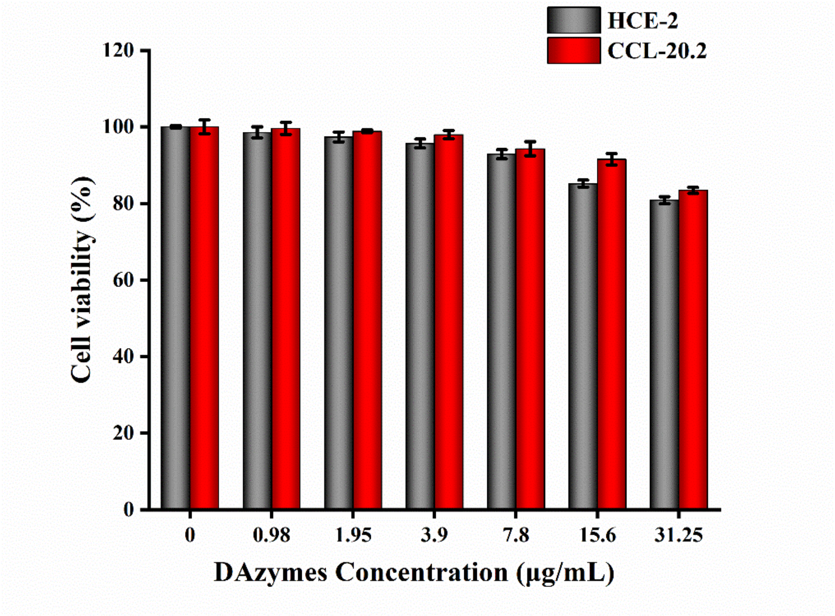

Fig. S10 Cytotoxicity of DAN in HCE-2 and CCL-20.2. In response to various concentrations of DAN (0, 0.98, 1.95, 3.9, 7.8, 15.6,31.25 μg/mL), the viabilities of HCE-2 and CCL-20.2 did not decrease obviously

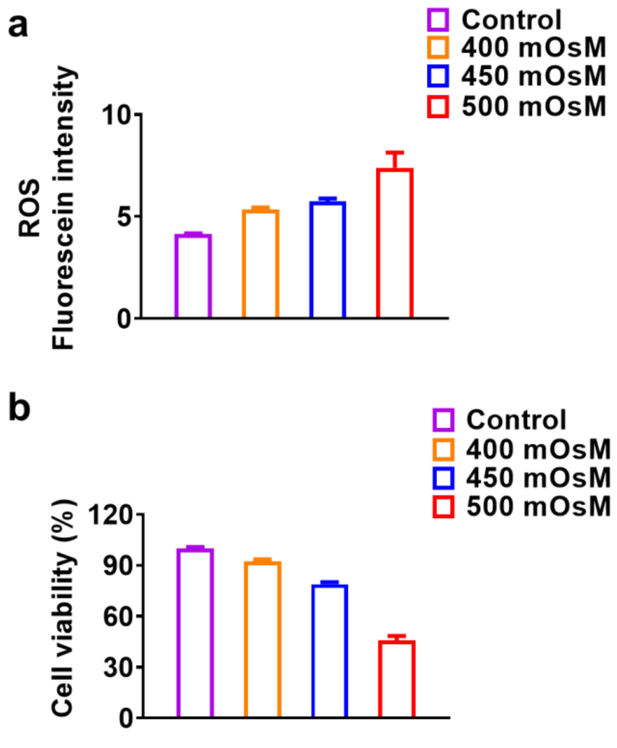

Fig. S11 **a** ROS production and **b** cell viability of HCE-2 cells treated with isotonic or hyperosmotic medium (400, 450 and 500 mOsM) for 24 h. The isotonic condition was used as a control

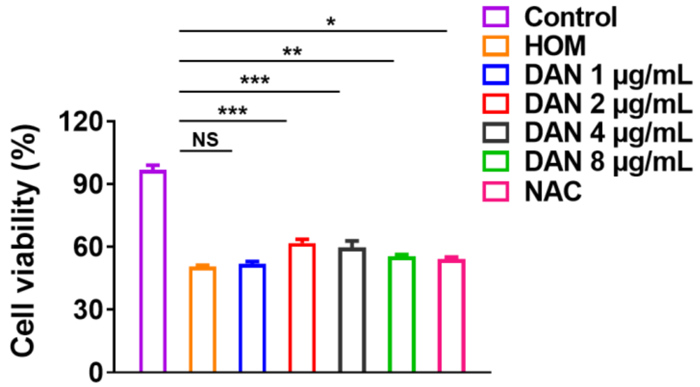

Fig. S12 Cell viability of HCE-2 cells exposure to hypertonic model (HOM, 500 mOsM) and pre-treated with various concentrations of DAN or NAC (10 mM). Data are presented as mean ± SD. $^{**}p < 0.01$, $^{***}p < 0.001$ and NS $p > 0.05$

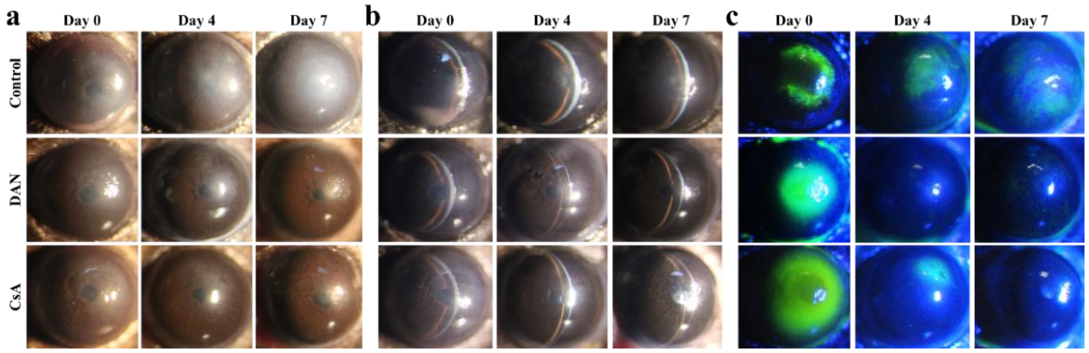

Fig. S13 a Optical, **b** slit-lamp and **c** corneal fluorescein-stained micrographs of mice eyes under different treatments

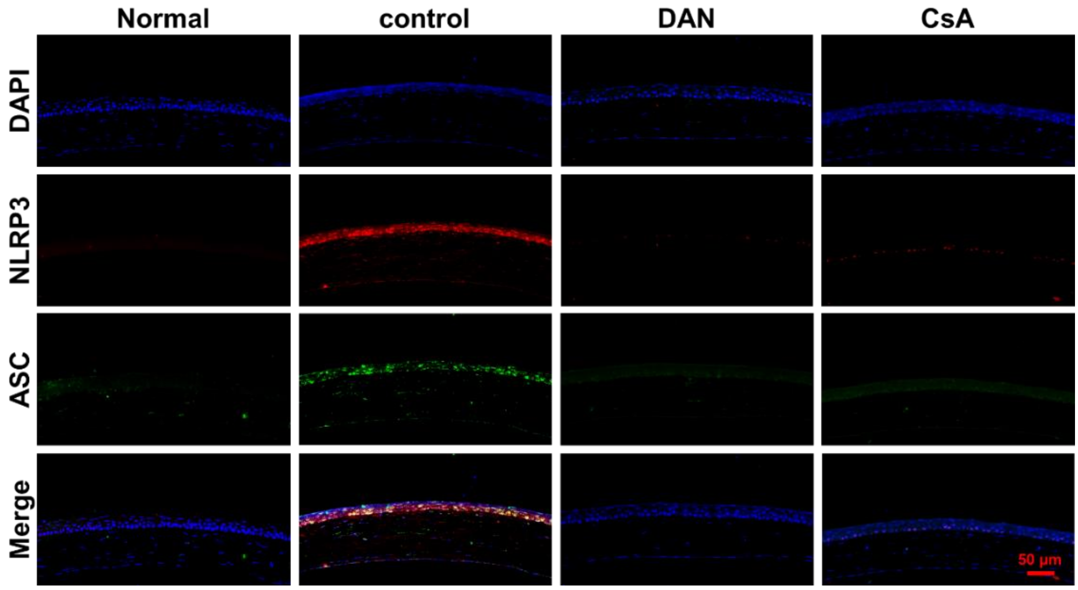

Fig. S14 Evaluations of NLRP3 and ASC expression were determined by immunofluorescence staining on the corneal epithelium in the normal and DED mice eyes after topical administration of saline, DAN, CsA. *Scale bar*: 50 μm

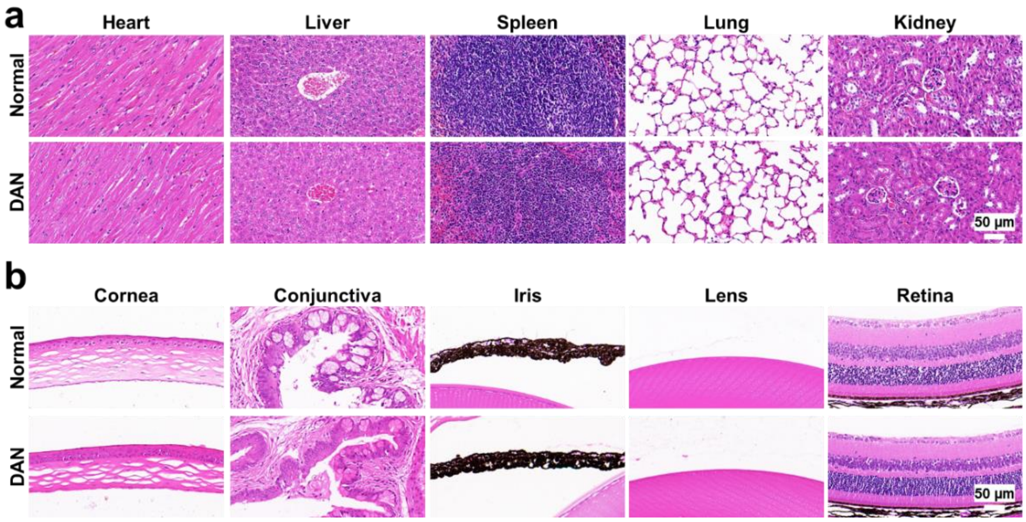

Fig. S15 *In vivo* biosafety assessment. H&E staining of histological sections of **a** heart, liver, spleen, lung, and kidney organs as well as **b** ocular tissues, including cornea, conjunctiva, iris, lens and retina after instilment with DAN twice per day for 7 days. *scale bar*: 50 μm

Supplementary References

[S1]B. Ravel, M. Newville, Athena, artemis, hephaestus: Data analysis for X-ray absorption spectroscopy using ifeffit. J. Synchrotron Rad. **12**, 537 (2005). https://doi.org/10.1107/s0909049505012719

[S2] S. I. Zabinsky, J. J. Rehr, A. Ankudinov, R. C. Albers, M. J. Eller, Multiple-scattering calculations of X-ray-absorption spectra. Phys. Rev. B **52**(4), 2995 (1995). https://doi.org/10.1103/physrevb.52.2995
